# RplI interacts with 5’ UTR of *exsA* to repress its translation and type III secretion system in *Pseudomonas aeruginosa*

**DOI:** 10.1371/journal.ppat.1010170

**Published:** 2022-01-05

**Authors:** Dan Wang, Xinxin Zhang, Liwen Yin, Qi Liu, Zhaoli Yu, Congjuan Xu, Zhenzhen Ma, Yushan Xia, Jing Shi, Yuehua Gong, Fang Bai, Zhihui Cheng, Weihui Wu, Jinzhong Lin, Yongxin Jin

**Affiliations:** 1 State Key Laboratory of Medicinal Chemical Biology, Key Laboratory of Molecular Microbiology and Technology of the Ministry of Education, Department of Microbiology, College of Life Sciences, Nankai University, Tianjin, China; 2 State Key Laboratory of Genetic Engineering, School of Life Sciences, Zhongshan Hospital, Fudan University, Shanghai, China; 3 Cancer Institute, the First Affiliated Hospital of China Medical University, Shenyang, China; Children’s Hospital Boston, UNITED STATES

## Abstract

*Pseudomonas aeruginosa* is an important opportunistic pathogen capable of causing variety of infections in humans. The type III secretion system (T3SS) is a critical virulence determinant of *P*. *aeruginosa* in the host infections. Expression of the T3SS is regulated by ExsA, a master regulator that activates the expression of all known T3SS genes. Expression of the *exsA* gene is controlled at both transcriptional and posttranscriptional levels. Here, we screened a *P*. *aeruginosa* transposon (Tn5) insertional mutant library and found *rplI*, a gene coding for the ribosomal large subunit protein L9, to be a repressor for the T3SS gene expression. Combining real-time quantitative PCR (qPCR), western blotting and *lacZ* fusion assays, we show that RplI controls the expression of *exsA* at the posttranscriptional level. Further genetic experiments demonstrated that RplI mediated control of the *exsA* translation involves 5’ untranslated region (5’ UTR). A ribosome immunoprecipitation assay and qPCR revealed higher amounts of a 24 nt fragment from *exsA* mRNA being associated with ribosomes in the Δ*rplI* mutant. An interaction between RplI and *exsA* mRNA harboring its 24 nt, but not 12 nt, 5’ UTR was confirmed by RNA Gel Mobility Shift and Microscale Thermophoresis assays. Overall, this study identifies the ribosomal large subunit protein L9 as a novel T3SS repressor that inhibits ExsA translation in *P*. *aeruginosa*.

## Author summary

Ribosomes provide all living organisms the capacity to synthesize proteins. The production of many ribosomal proteins is often controlled by an autoregulatory feedback mechanism. *P*. *aeruginosa* is an opportunistic human pathogen and its type III secretion system (T3SS) is a critical virulence determinant in host infections. In this study, by screening a Tn5 mutant library, we identified *rplI*, encoding ribosomal large subunit protein L9, as a novel repressor for the T3SS. Further exploring the regulatory mechanism, we found that the RplI protein interacts with the 5’ UTR (5’ untranslated region) of *exsA*, a gene coding for transcriptional activator of the T3SS. Such an interaction likely blocks ribosome loading on the *exsA* 5’ UTR, inhibiting the initiation of *exsA* translation. The significance of this work is in the identification of a novel repressor for the T3SS and elucidation of its molecular mechanism. Furthermore, this work provides evidence for individual ribosomal protein regulating mRNA translation beyond its autogenous feedback control.

## Introduction

*P*. *aeruginosa* is an important opportunistic pathogen capable of causing a variety of infections in humans, particularly in patients with burn wounds, immunodeficiency and cystic fibrosis [1]. *P*. *aeruginosa* utilizes multifactorial virulence properties to establish acute and chronic infections. The type III secretion system (T3SS) is a critical virulence determinant of *P*. *aeruginosa* in acute infections [2]. Deficiency in T3SS gene expression/function severely attenuates *P*. *aeruginosa* virulence in mouse infection models of acute pneumonia, burn wounds, corneas, and neutropenia [3–6].

In *P*. *aeruginosa*, the T3SS can be induced by growth in a low Ca^2+^ environment or direct contact with host cells [3]. ExsA, an AraC-type DNA binding protein, is the master regulator of T3SS that can activate all of the known T3SS genes, including its own operon through the P_*exsC*_ promoter [3]. Many regulatory factors have been determined to control the expression of T3SS through direct or indirect regulation of the *exsA* transcription and/or translation [3]. In addition to the *exsC* promoter (P_*exsC*_), transcription of the *exsA* is also driven by its own promoter P_*exsA*_, whose transcriptional activity is much lower than that of the P_*exsC*_ [7]. Unlike P_*exsC*_ which is directly bound and activated by ExsA, the P_*exsA*_ promoter is directly bound and stimulated by the cAMP-Vfr signaling system [7]. Once transcribed, the *exsA* mRNA is subject to regulatory control at the posttranscriptional level. Translation of the *exsA* is positively controlled by the RNA helicase DeaD and the RNA-binding protein RsmA, but negatively regulated by a small noncoding RNA, sRNA 0161 [8–11].

In the present report, we identified *rplI* as a repressor for expression of the T3SS in *P*. *aeruginosa* through a genetic screen for isolates with altered T3SS expression in the mPAO1 strain. We show that mutants lacking *rplI* have increased expression of the T3SS and cytotoxicity toward cultured mammalian cells. We demonstrate that RplI controls expression of the T3SS through repressing the ExsA translation. Further studies revealed that RplI mediated translational repression requires a 24-nucleotide fragment residing upstream of the *exsA* coding region. EMSA and MST assays demonstrate that RplI can bind to the 24 nt upstream of the *exsA* start codon. In combination, these data identified a novel posttranscriptional regulatory mechanism that negatively controls the *P*. *aeruginosa* T3SS gene expression, elucidating a novel function of the L9 ribosomal protein in gene repression other than feedback control.

## Results

### Isolation of mutants with elevated P_*exsC*_ expression

**Fig 1:**
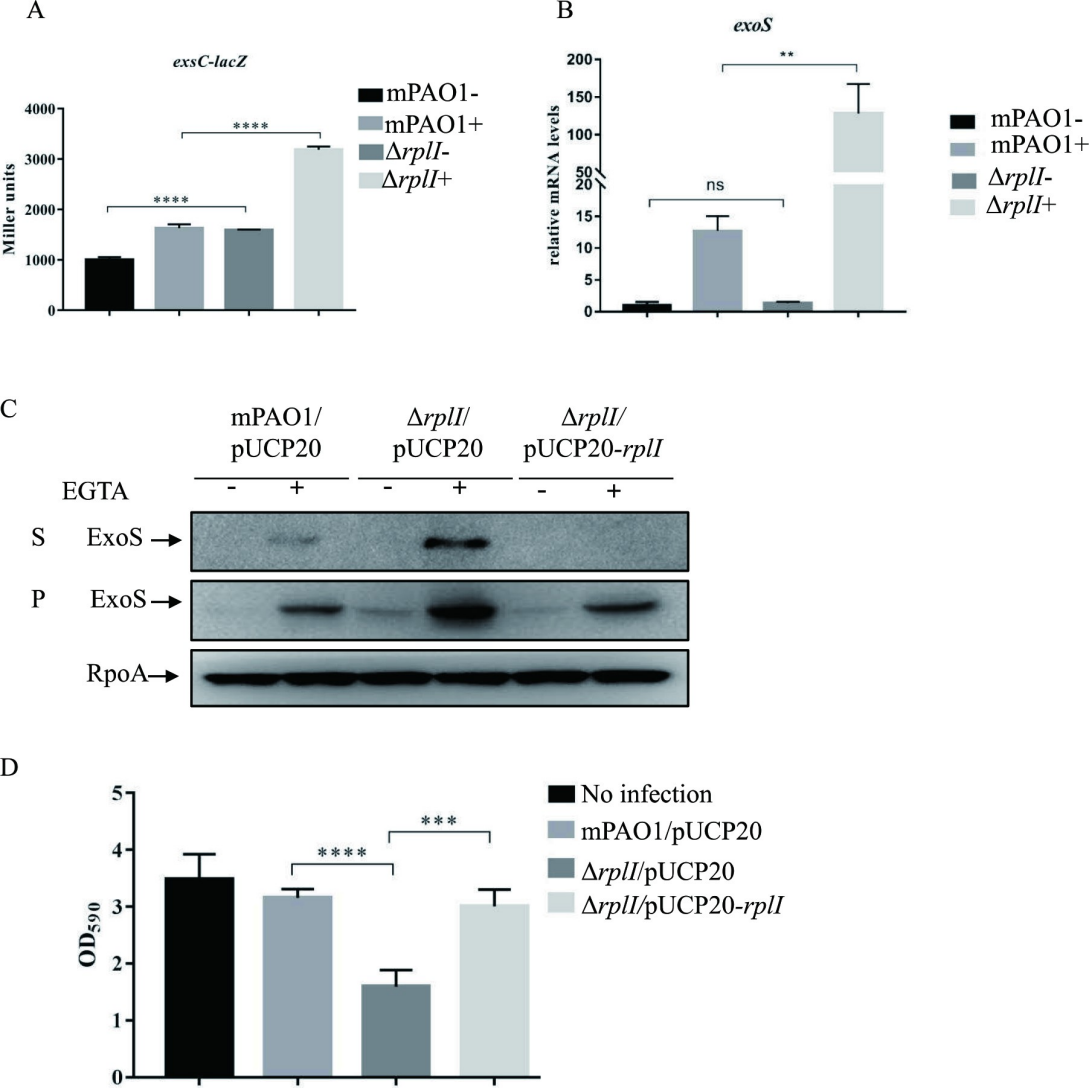
RplI represses T3SS expression and cytotoxicity. (A) mPAO1 and Δ*rplI* containing the P_*exsC*_-*lacZ* transcriptional fusion were grown to an OD_600_ of 1.0 in LB with (+) or without (-) 5 mM EGTA and subjected to β-galactosidase assays. Each assay was performed in triplicate, and the error bars indicate standard deviations. ****P < 0.0001 compared with wild-type mPAO1 by Student’s *t* test. (B) Relative *exoS* mRNA levels in the mPAO1 and Δ*rplI* strains. Total RNA was isolated under T3SS inducing (+) and non-inducing (-) conditions with 5 mM EGTA, and the *exoS* mRNA levels were determined by real-time qPCR using *rpsL* as the internal control. ns, not significant, **P < 0.01, by Student’s *t* test. (C) Expression and secretion of ExoS in mPAO1/pUCP20, Δ*rplI*/pUCP20 and Δ*rplI*/pUCP20*-rplI*. Bacteria were cultured to an OD_600_ of 1.0 in LB medium with or without 5 mM EGTA. Proteins in supernatants (S) and pellets (P) from equivalent bacterial cells were loaded onto 12% SDS-PAGE gels and probed with an antibody against ExoS or an anti-RpoA antibody. The data shown represent the results from three independent experiments. (D) Cytotoxicity of mPAO1/pUCP20, Δ*rplI*/pUCP20 and Δ*rplI*/pUCP20*-rplI*. HeLa cells were infected with the indicated strains at an MOI of 50. Three hours post infection, cells attached to the 24-well plate were washed with PBS and stained with crystal violet. The cell-associated crystal violet was dissolved in ethanol and quantified by measuring the OD_590_. HeLa cells with no bacterial infection served as a control. ***P< 0.001, ****P < 0.0001 by Student’s *t* test.

To identify novel repressors of the T3SS in *P*. *aeruginosa*, a Tn5 transposon insertion library was generated in mPAO1 strain containing a P_*exsC*_-*lacZ* reporter plasmid. The mPAO1 displays a lower level expression of T3SS compared to PAK strain due to *mexT* gene mutation and was referred as PAO1 in our previous study [12]. Approximately 10, 000 mutants were screened for dark blue colonies on X-gal plates under Ca^2+^ depletion conditions. A total of 34 transposon mutants displayed darker blue color compared to the parental mPAO1/P_*exsC*_-*lacZ* strain. These mutants were then subjected to sequence analysis to locate the Tn5 insertion sites. Total 8 different genetic loci on the chromosome were found to have the Tn5 insertions (S1 Table). Of them, 2 mutants had Tn5 insertions at two sites (372 bp/447 bp) on a *rplI* gene, which encodes 50S ribosomal protein L9. A *rplI* gene deletion mutant was further generated in the background of mPAO1 and introduced with the P_*exsC*_-*lacZ* reporter plasmid. Liquid β-galactosidase assay results confirmed that the *rplI* mutant indeed displays an elevated β-galactosidase activity (Fig 1A).

### RplI represses ExoS expression and reduces bacterial cytotoxicity

We further pursued the role of RplI in the regulation of the T3SS. RplI encodes an L9 protein of the ribosomal 50S large subunit and the *rplI* deletion mutant displayed no discernible growth rate change when cultured in LB medium (S1A Fig), which suggested that L9 is not essential in *P*. *aeruginosa*, similar to that in *Salmonella enterica* [13]. Consistent with the phenotypes of the original transposon insertion mutant, the mPAO1Δ*rplI* mutant showed higher mRNA levels of *exoS*, *pcrV* and *pscF* (Figs 1B and S1B), as well as increased expression and secretion of ExoS (Fig 1C) compared to those in the wild type mPAO1. Complementation with a *rplI*-expressing plasmid decreased the expression and secretion of ExoS (Fig 1C). To further confirm the relationship between *rplI* and T3SS, the *rplI* gene was deleted in another PAO1 laboratory strain which has a proficient T3SS. Similar to Δ*rplI* in mPAO1, this mutant also displayed increased expression and secretion of ExoS compared to the parental wild type PAO1, which could be complemented by *rplI* expression in trans (S1C Fig). T3SS-mediated cytotoxicity was further measured by crystal violet staining assay. When HeLa cells were infected with wild-type mPAO1/pUCP20 at an MOI (multiplicity of infection) of 50, majority of the cells looked fine and remained attached after 3 hours of infection. Loss of the *rplI*, however, resulted in a strain that was much more cytotoxic, and complementation with a *rplI* gene decreased the cytotoxicity (Fig 1D). These results suggested that RplI is a repressor of the T3SS in *P*. *aeruginosa*.

### Role of RplI in the expression of *exsA* gene

**Fig 2:**
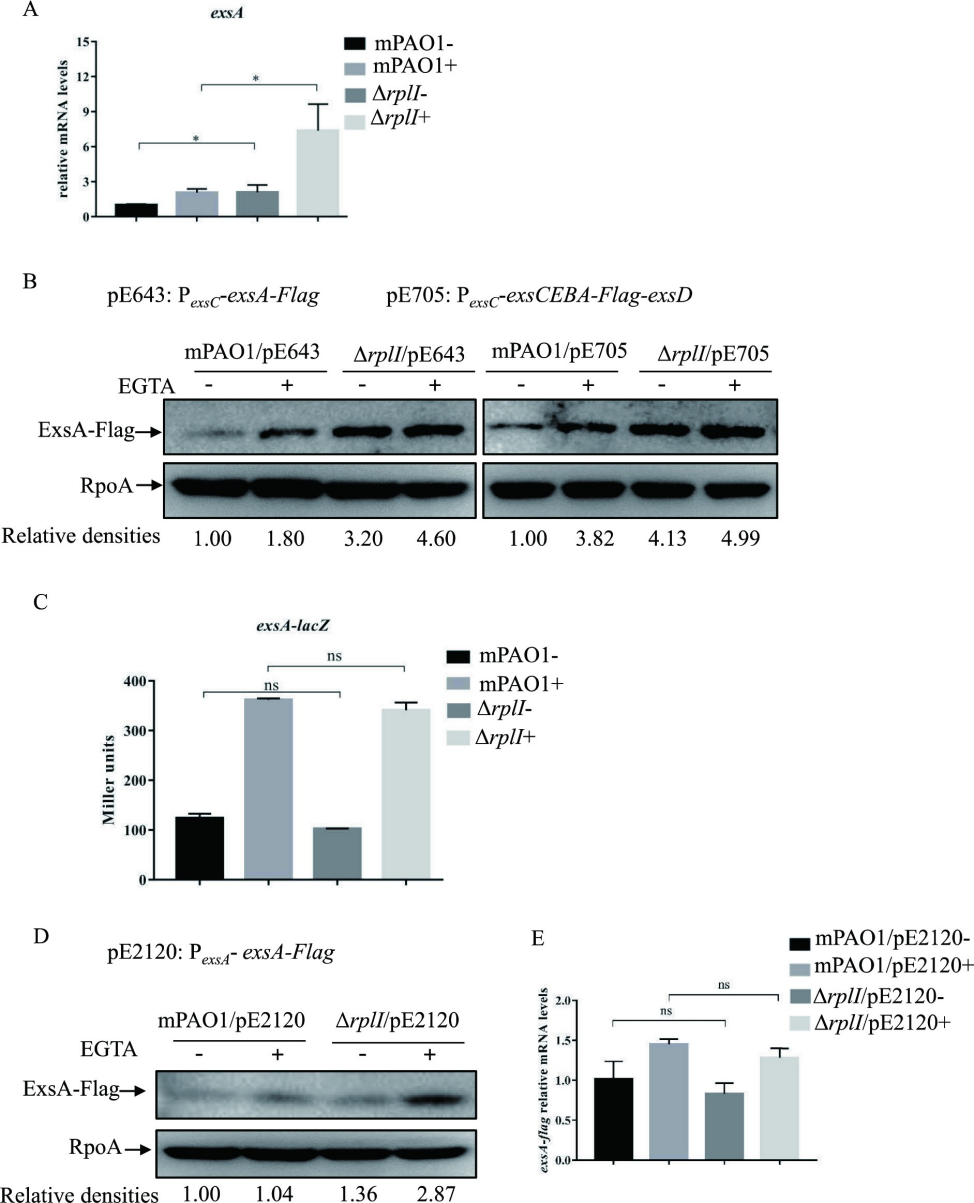
Role of RplI in the expression of ExsA. (A and E) The relative *exsA* (A) or *exsA-flag* (E) mRNA levels in mPAO1 and Δ*rplI* containing pE2120 (E) or not (A). Total RNA was isolated under T3SS inducing (+) and non-inducing (-) conditions with 5 mM EGTA and the *exsA* or *exsA-flag* mRNA levels were determined by real-time qPCR using *rpsL* as the internal control. ns, not significant, *P < 0.05, by Student’s *t* test. (B and D) Amounts of ExsA-Flag. mPAO1 and Δ*rplI* containing pE643 or pE705 integrated into the chromosome or pE2120 were grown to an OD_600_ of 1.0 in LB with (+) or without (-) 5 mM EGTA and collected by centrifugation. Samples from equivalent numbers of bacterial cells were separated by SDS-PAGE and probed with an anti-Flag or an anti-RpoA antibody. The density of each band was determined with Image J. Relative densities represent the density of ExsA-Flag/density of RpoA with the respective first lane as 1. The data shown represent the results from three independent experiments. (C) mPAO1 and Δ*rplI* containing the P_*exsA*_-*lacZ* transcriptional fusion were grown to an OD_600_ of 1.0 in LB with (+) or without (-) 5 mM EGTA and subjected to β-galactosidase assays. Each assay was performed in triplicate, and the error bars indicate standard deviations. ns, not significant compared with wild-type mPAO1 by Student’s *t* test.

ExsA is the central activator of the T3SS located in the *exsCEBA* operon [3]. We wanted to know whether the significant increase in ExoS expression in the Δ*rplI* mutant was due to the upregulation of *exsA* expression. Real-time qPCR was performed to examine the *exsA* mRNA level in mPAO1 and its Δ*rplI* mutant. Consistent with the increased *exsC* promoter activity (Fig 1A), the mRNA level of *exsA* in the Δ*rplI* mutant was significantly higher than that in wild type mPAO1 (Fig 2A). To further confirm the increased expression of ExsA protein, a C-terminal Flag-tagged ExsA (alone/pE643 or in the *exsCEBA* operon/pE705) [14] driven by the *exsC* promoter was integrated into the chromosome of *P*. *aeruginosa*. Consistent with the elevated *exsA* transcription, the Δ*rplI* mutant displayed an increased ExsA-Flag protein level under both T3SS inducing and non-inducing conditions (Figs 2B, S2A, and S2B). These results indicated that RplI directly or indirectly represses the expression of the *exsCEBA* operon.

ExsA is known to activate its own expression through the promoter of *exsC* operon [15]; therefore, the upregulation of *exsA* expression in the Δ*rplI* mutant might occur at either transcriptional or posttranscriptional level. To address this, a P_*exsA*_-*lacZ* transcriptional fusion reporter plasmid [16] was introduced into the wild type mPAO1 and its Δ*rplI* mutant. In both strains, the β-galactosidase activities were significantly higher under T3SS inducing condition than that under non-inducing condition. However, mRNA levels displayed no difference between wild type mPAO1 and the Δ*rplI* mutant under either T3SS inducing or non-inducing condition (Fig 2C). To visualize the ExsA protein expression, pE2120 encoding a C-terminal Flag-Tagged ExsA driven by its own promoter (the promoter of *exsA*) [16], was introduced into the two bacterial strains. Consistent with the results of P_*exsA*_-*lacZ* transcriptional fusion, the relative mRNA levels of *exsA-flag* (primers shown in S3 Table) showed no difference in the Δ*rplI* mutant compared to that in wild type mPAO1 strain. However, an increased ExsA-Flag protein level was observed in the Δ*rplI* mutant compared to that in the wild type mPAO1 strain (Fig 2D and 2E). These data suggested that RplI likely affects the ExsA expression at the posttranscriptional level.

### RplI controls the expression of ExsA at the posttranscriptional level

**Fig 3:**
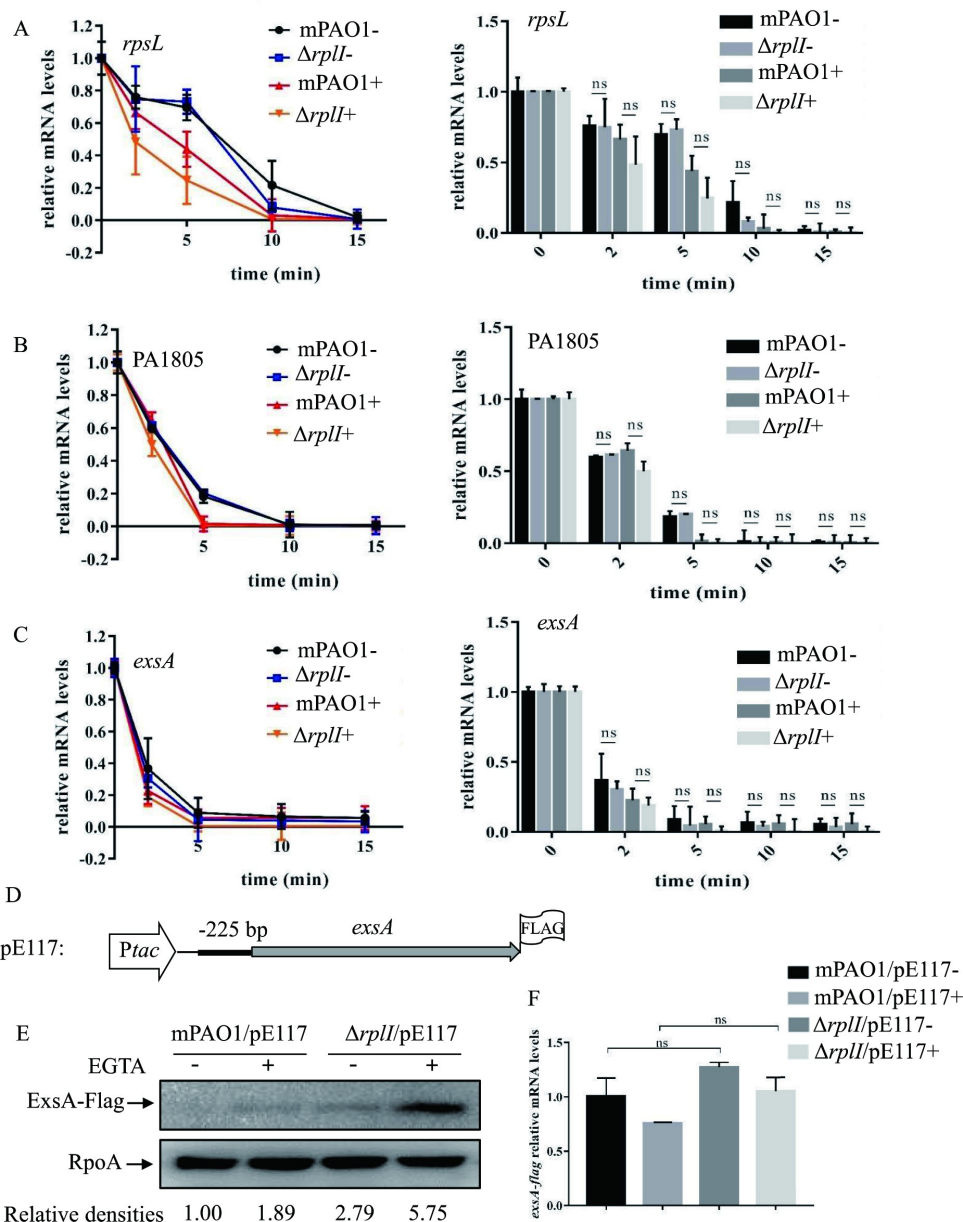
RplI controls the expression of ExsA at the posttranscriptional level by repressing ExsA translation. (A-C) Degradation of *rpsL* (A), PA1805 (B), and *exsA* (C) mRNA in wild type mPAO1 and its Δ*rplI* mutant under T3SS inducing (+) and non-inducing (-) conditions with 5 mM EGTA. Bacterial cells were treated with rifampicin, collected at 2, 5, 10, and 15 min and mixed with equal numbers of *gfp-*expressing *E*. *coli* cells. Total RNA was purified, and the relative mRNA levels were determined by real-time qPCR. The *gfp* mRNA level in each sample was used as the internal control for normalization. The right panel is the bar diagram of the respective left panel. ns, not significant by Student’s *t* test. (D) Construct of *exsA*-Flag in pE117. (E) Amounts of ExsA-Flag. mPAO1 and Δ*rplI* containing pE117 were grown to an OD_600_ of 1.0 in LB with (+) or without (-) 5 mM EGTA and collected by centrifugation. Samples from equivalent numbers of bacterial cells were separated by SDS-PAGE and probed with an anti-Flag or an anti-RpoA antibody. The density of each band was determined with Image J. Relative densities represent the density of ExsA-Flag/density of RpoA with the first lane as 1. The data shown represent the results from three independent experiments. (F) Relative *exsA-flag* mRNA levels in mPAO1 and Δ*rplI* containing pE117. Total RNA was isolated under T3SS inducing (+) and non-inducing (-) conditions with 5 mM EGTA, and the *exsA-flag* mRNA levels were determined by real-time qPCR using *rpsL* as the internal control. ns, not significant, by Student’s *t* test.

The higher protein level of ExsA in the Δ*rplI* mutant might be due to an increased *exsA* mRNA stability or enhanced *exsA* translation. To address these possibilities, we initially compared the stability of *exsA* mRNA in wild type mPAO1 and the Δ*rplI* mutant. Bacteria were cultured to an OD_600_ of 1.0 under either T3SS inducing or non-inducing condition, and 200 μg/mL rifampicin was added to inhibit further transcription. After 2, 5, 10 or 15 min, total RNAs were collected and examined with real-time qPCR (primers in S3 Table). As shown in Fig 3, the *exsA* mRNA decay rates displayed no significant difference between mPAO1 and its Δ*rplI* mutant derivative under either T3SS-inducing or non-inducing condition. These data indicated that the increased ExsA protein level was not due to changes in the *exsA* mRNA stability.

Next, we examined whether translation of the ExsA was enhanced in the Δ*rplI* mutant using a previously constructed *exsA*-Flag fusion plasmid (*exsA*-Flag-S/pE117) [17]. In this plasmid, the *exsA* coding sequence with its 225-bp upstream region was driven by a *tac* promoter, and a Flag-tag was fused at the C-terminus of the ExsA (Fig 3D). Western blot assay revealed an increased ExsA-Flag level in the Δ*rplI* mutant compared to that in wild type mPAO1 containing the pE117 (Figs 3E and S2C), while the mRNA levels of *exsA-flag* displayed no difference between the two strains (Fig 3F). These results suggested that RplI represses ExsA translation.

### RplI-dependent repression of the ExsA translation requires a -24 bp region of the *exsA* 5’ UTR

**Fig 4:**
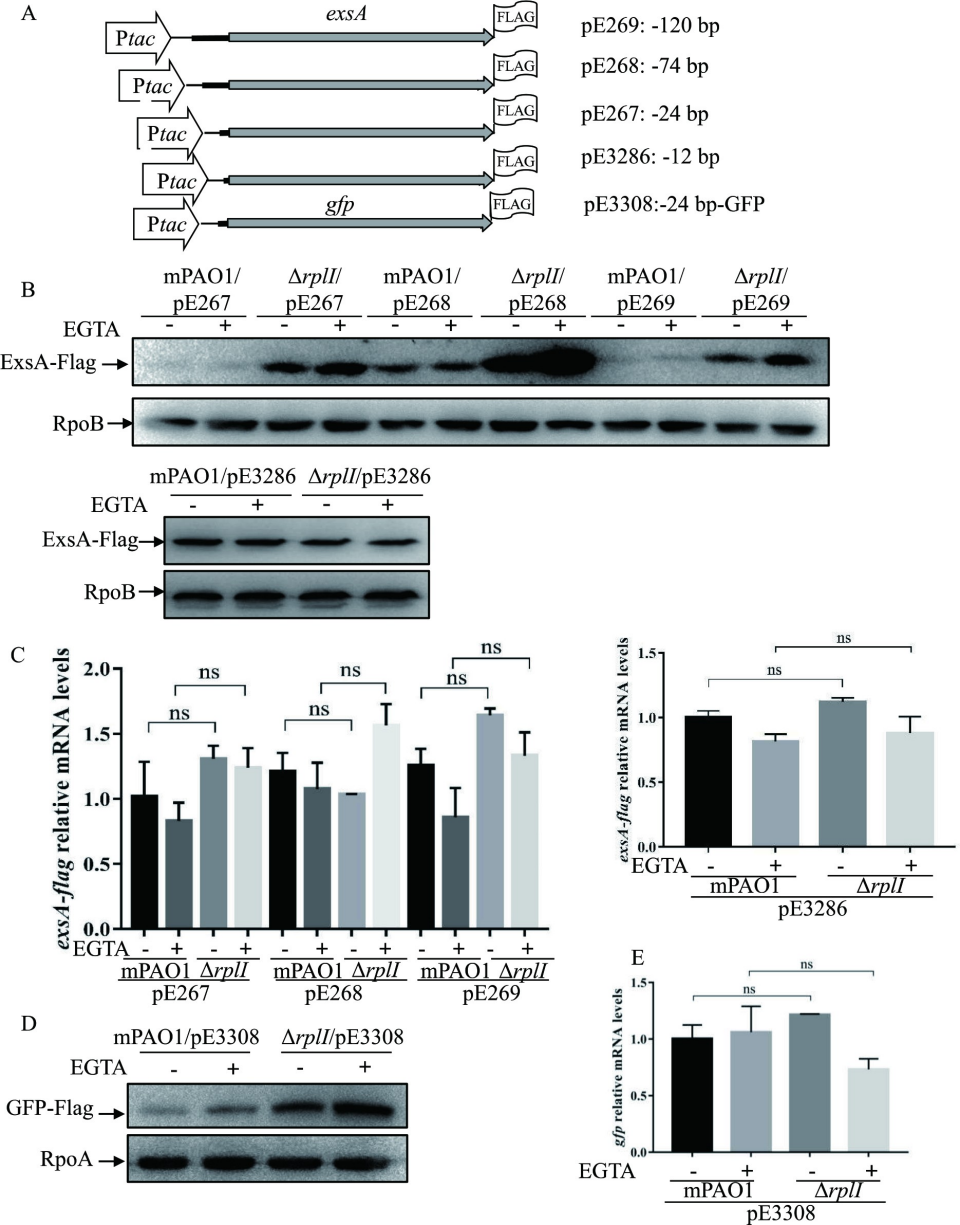
RplI-dependent repression of ExsA translation requires a 24 bp 5’ untranslated region of *exsA*. (A) Constructs of *exsA*-Flag and *gfp*-Flag. (B and D) Amounts of ExsA-Flag or GFP-Flag in the indicated strains. Overnight-cultured mPAO1 and Δ*rplI* containing the indicated constructs were diluted 1:50 into LB medium, grown to an OD_600_ of 1.0 with (+) or without (-) 5 mM EGTA and collected by centrifugation. Samples from equivalent numbers of bacterial cells were loaded onto an SDS-PAGE gel and probed with an anti-Flag, an anti-RpoB antibody or an anti-RpoA antibody. The data shown represent the results from three independent experiments. (C and E) *exsA-flag* (C) or *gfp* (E) mRNA levels in mPAO1 and Δ*rplI* containing pE267, pE268, pE269 or pE3286 (C) or pE3308 (E). Total RNA was isolated under T3SS inducing (+) and non-inducing (-) conditions with 5 mM EGTA, and the *exsA-flag* or *gfp* mRNA levels were determined by real-time qPCR using *rpsL* as the internal control. ns, not significant, by Student’s *t* test.

Based on our finding that RplI attenuates ExsA translation, we wanted to know the sequence requirements for the RplI-mediated control. Plasmid constructs, carrying the *tac* promoter driven *exsA-flag* fusion gene with varying lengths of the 5’ untranslated region (5’ UTR) of *exsA*, were introduced into the mPAO1 and Δ*rplI* strains (Fig 4A). The mRNA levels and protein amounts of ExsA-Flag were examined with real-time qPCR and Western blot analysis, respectively. As shown in Fig 4B and 4C, in the presence of -120, -74, and -24 bp upstream regions, despite of comparative mRNA levels, ExsA-Flag amounts were much higher in the Δ*rplI* mutant than those in the wild type mPAO1. In contrast, pE3286, which harbors the *exsA-Flag* and its -12 bp upstream region, rendered mPAO1 and Δ*rplI* similar amounts of ExsA-Flag. These data suggested that while most of the sequence upstream of the *exsA* ORF is dispensable for the RplI-mediated regulation, at least a 24-nt untranslated upstream sequence is required.

Next, we determined whether the *exsA* coding sequence was required. The *exsA* coding sequence in pE267 was replaced with a *gfp* gene, resulting in pE3308, in which the *gfp* coding region was fused with the 24-bp 5’ UTR sequence of *exsA* (Fig 4A). Similar to the pE267 (P_*tac*_-24-*exsA*-Flag), *gfp-flag* fusion with the 24 bp 5’ UTR of *exsA* resulted in a higher GFP-Flag protein level in the Δ*rplI* mutant than that in wild type mPAO1, although their mRNA levels for *gfp* were similar (Fig 4D and 4E). This finding suggested that the 24 bp upstream region of the *exsA* coding sequence plays a critical role in the RplI-mediated translational repression of ExsA.

### Enhanced ribosome binding to the *exsA* mRNA in the Δ*rplI* mutant

**Fig 5:**
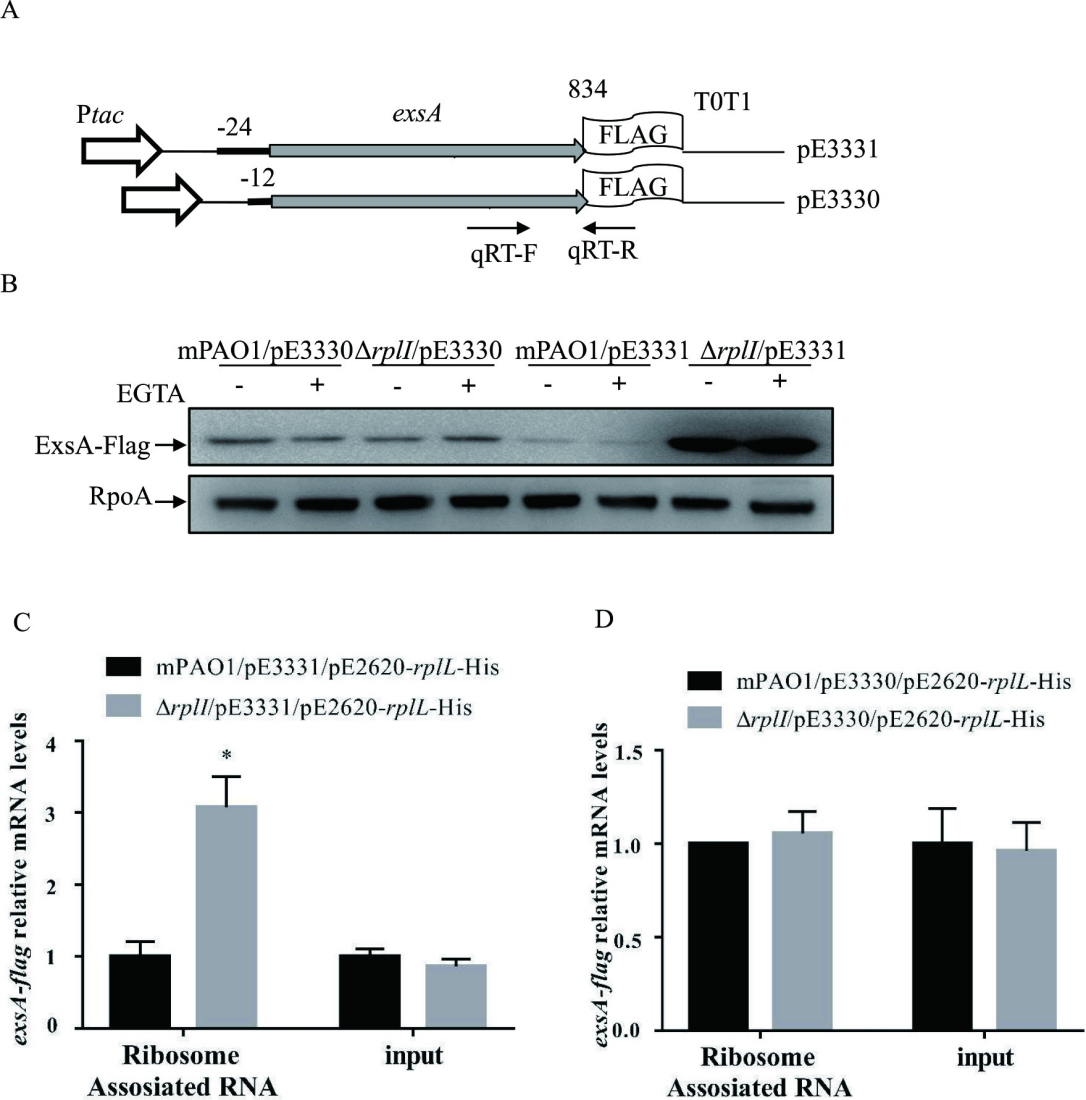
Expression of ExsA-Flag and quantification of ribosome-associated *exsA* mRNAs. (A) Constructs of P_*tac*_-24 bp-*exsA*-Flag-T0T1 (pE3331) and P_*tac*_-12 bp-*exsA*-Flag-T0T1 (pE3330). The positions of qPCR primers are represented by arrows. (B) The protein levels of ExsA-Flag in the indicated strains. mPAO1 and Δ*rplI* containing pE3330 or pE3331 were grown to an OD_600_ of 1.0 in LB with (+) or without (-) 5 mM EGTA and collected by centrifugation. Samples from equivalent numbers of bacterial cells were loaded onto an SDS-PAGE gel and probed with an anti-Flag or an anti-RpoA antibody. The data shown represent the results from three independent experiments. (C and D) mPAO1 and Δ*rplI* containing pE2620-*rplL*-His and pE3331 (C) or pE3330 (D) were lysed with sonication and subjected to Ni-NTA chromatography or not (input), followed by RNA purification. The relative mRNA levels of *exsA*-Flag were determined by real-time qPCR with 16S ribosome RNA (PA0668.1) as the internal control. *, *P* < 0.05 by Student’s *t* test.

Based on our finding that ExsA translation is increased in a Δ*rplI* mutant and the fact that *rplI* encodes the L9 protein of the 50S subunit of ribosomes, we hypothesized that more ribosomes might associate with the 24 nt 5’ UTR of *exsA* in the absence of RplI. To test this possibility, a modified RNA-binding protein immunoprecipitation was coupled with real-time qPCR to directly determine the amount of ribosome-associated *exsA* mRNA as previously described [18]. First, using pE267 plasmid as a template, we cloned the *exsA-flag* with its 24 bp 5’ UTR together with the *tac* promoter and two consecutive transcription terminators (*tac* promoter and T0T1 from pFlag-CTC, Sigma) into pE1553 [16], resulting in pE3331 (Fig 5A). The RNA transcribed from this construct harbors a single ribosome binding site for *exsA*; thus, ribosomes can only bind to the *exsA* mRNA. The C-terminal Flag ensures that *exsA* mRNA transcribed from the genomic locus will not be detected by real-time qPCR (primer set shown in Fig 5A). Indeed, no amplicon could be observed when the detection primer set (S3 Table) was used with mPAO1 genomic DNA as template in the PCR. A control plasmid pE3330, in which the 24-bp 5’ UTR was replaced by a 12-bp 5’ UTR, was also constructed (Fig 5A). The ExsA-Flag amounts were then examined in mPAO1 and the Δ*rplI* mutant containing pE3331 or pE3330. As the results shown in Fig 5B, similar to pE267 and pE3286, the 24 nt 5’ UTR of *exsA* rendered an increased amount of ExsA-Flag protein in Δ*rplI*, while the 12 nt 5’ UTR of *exsA* displayed similar amounts of ExsA-Flag between wild type mPAO1 and the Δ*rplI* strain.

A plasmid construct with another 50S ribosomal protein L7/L12 coding gene *rplL* fused with *his-tag* (pE2620-*rplL*-His) was further introduced into the wild type mPAO1 and the Δ*rplI* mutant containing either pE3330 or pE3331. Ribosomes were then purified from the cell extracts of these strains by Ni-NTA chromatography, and the associated RNA was isolated and subjected to real-time qPCR assay. The mRNA levels of *exsA* transcribed from both plasmids were similar between mPAO1 and the Δ*rplI* mutant (Fig 5C and 5D). However, many more *exsA* mRNAs with 24 nt 5’ UTR, but not with 12 nt 5’ UTR, were associated with the ribosome isolated from the Δ*rplI* mutant (Fig 5C and 5D). In combination, these results suggested that increased amounts of ribosomes were associated with the 24-nt 5’ UTR of *exsA* in the Δ*rplI* mutant.

### Sr0161 is not involved in the RplI mediated control of ExsA translation

**Fig 6:**
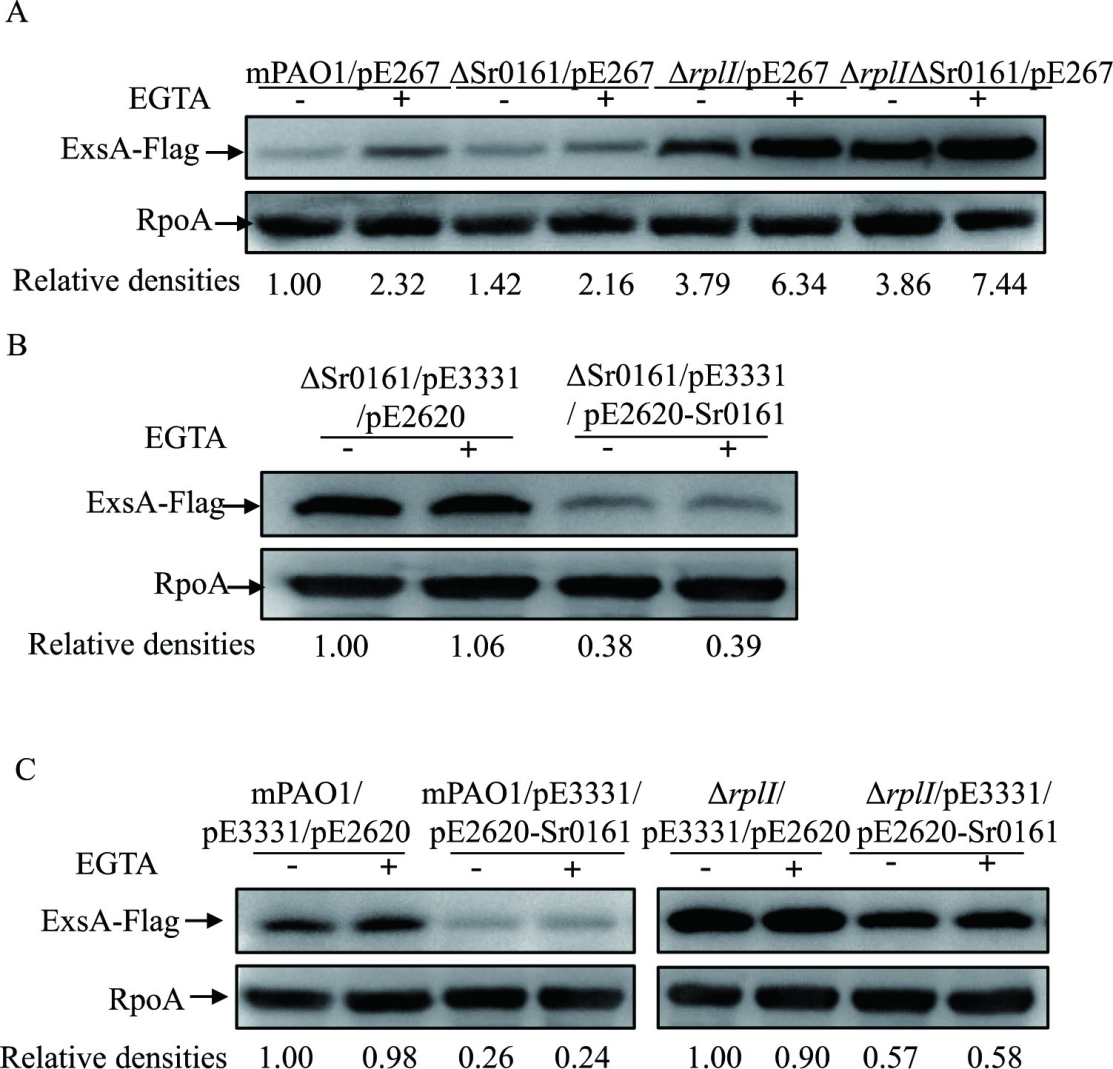
Sr0161 is not involved in the RplI mediated control of ExsA translation. The protein levels of ExsA-Flag in mPAO1, ΔSr0161, Δ*rplI* and Δ*rplI*ΔSr0161 containing pE267 (A); ΔSr0161 containing pE3331 and empty vector (pE2620) or pE2620-Sr0161 (B); mPAO1 and Δ*rplI* containing pE3331 and empty vector (pE2620) or pE2620-Sr0161 (C). Bacteria cultured overnight were diluted 1:50 into LB medium with 1 mM IPTG (B and C) or without IPTG (A), grown to an OD_600_ of 1.0 with (+) or without (-) 5 mM EGTA, and collected by centrifugation. Samples from equivalent numbers of bacterial cells were separated by SDS-PAGE and probed with an anti-Flag or an anti-RpoA antibody. The density of each band was determined with Image J. Relative densities represent the density of ExsA-Flag/density of RpoA with the respective first lane as 1. The data shown represent the results from three independent experiments.

A recent study showed that a small regulatory RNA Sr0161 interacts with the 5’ UTR of *exsA* mRNA to repress ExsA synthesis at the posttranscriptional level, and overexpression of Sr0161 decreased the expression of the T3SS in *P*. *aeruginosa* [9,10]. The 5’ UTR of *exsA*, between -24 nt and -14 nt, was predicted to base pair with Sr0161 [9,10]. Because 24 bp, but not 12 bp, 5’ UTR of *exsA* was needed for the RplI-mediated translational repression of *exsA*, we tested whether Sr0161 was also involved in the RplI mediated control of ExsA translation. The Sr0161 gene was deleted in mPAO1 and the Δ*rplI* mutant backgrounds, and then pE267 (-24 bp-*exsA*-Flag construct) was introduced into the resulting mutant strains. Consistent with a previous report [9], no increase in ExsA-Flag protein level was observed in the ΔSr0161 compared to that of mPAO1 (Fig 6A). Similarly, the amount of ExsA-Flag in Δ*rplI* was the same as that in Δ*rplI*ΔSr0161.

To further test the hypothesis, we examined the influence of Sr0161 overexpression on the expression of ExsA. We cloned Sr0161 into pE2620 (pMMB67EH, Gm) and introduced it into ΔSr0161 harboring pE3331 (-24 bp-*exsA*-Flag). Consistent with the previous study [9], overexpression of Sr0161 reduced the amounts of ExsA-Flag in ΔSr0161 (Fig 6B). Next, the pE2620-Sr0161 and -24 bp-*exsA*-Flag (pE3331) were co-introduced into mPAO1 and Δ*rplI*. Similar to that in ΔSr0161, overexpression of the Sr0161 decreased the amount of ExsA-Flag in the wild type mPAO1 strain (Fig 6C). Again, the reduced amount of ExsA-Flag was observed in Δ*rplI* with overexpression of the Sr0161 (Fig 6C). Together, these data indicated that Sr0161 was not involved in the RplI mediated repression of the ExsA.

### RplI binds to the 24 nt 5’ UTR of the *exsA* mRNA

**Fig 7:**
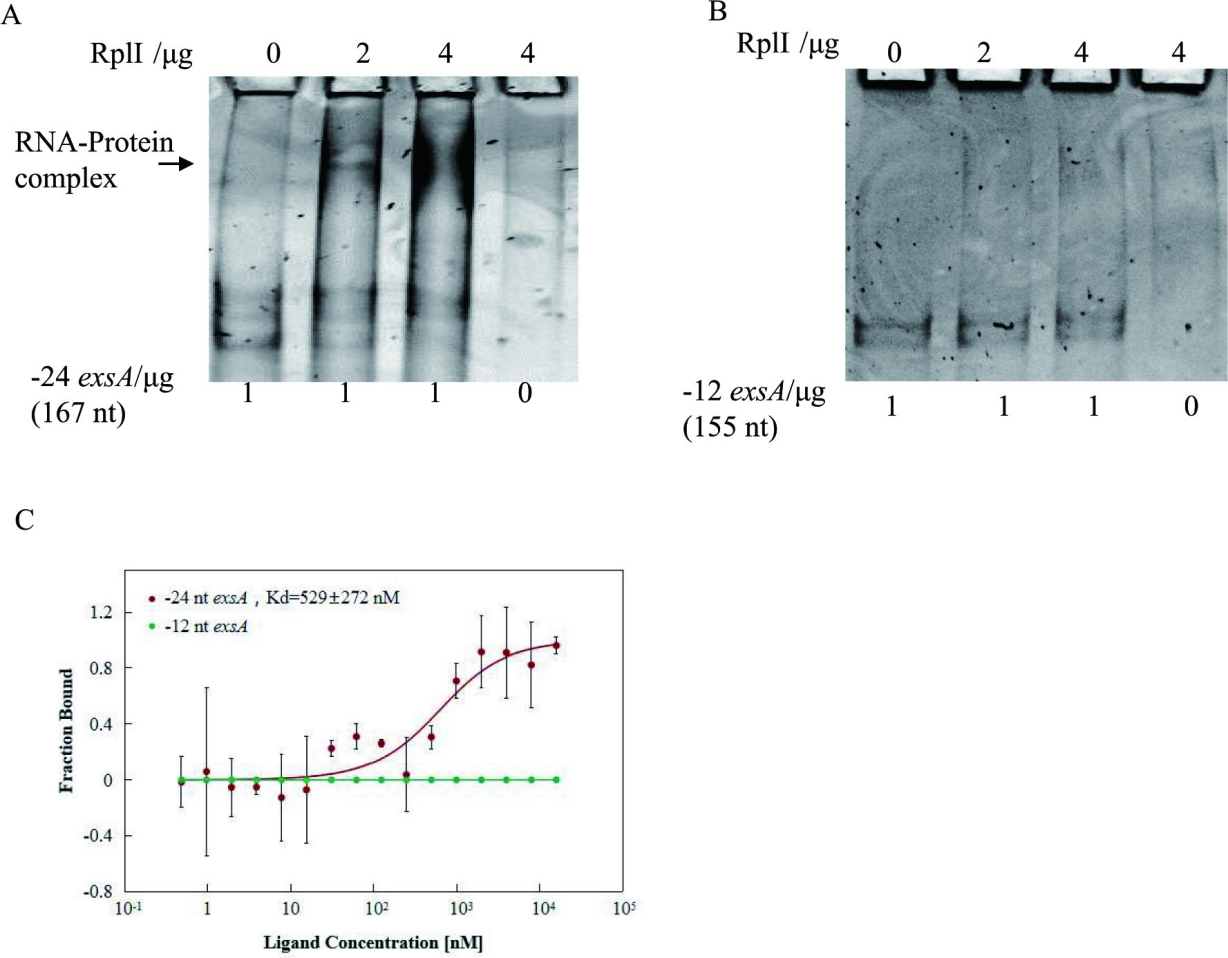
RplI interacts with *exsA* mRNA containing a 24 nt 5’ UTR but not a 12 nt 5’ UTR. (A and B) Binding of RplI to the indicated RNAs was examined by EMSA. Increasing amounts of purified *P*. *aeruginosa* RplI-His were incubated with 167 nt/155 nt (A/B) *exsA* mRNA respective corresponding to the 24 nt/12 nt 5’ UTR and its first 143 nt mRNA on ice for 30 min. The mixtures were electrophoresed on an 8% native polyacrylamide gel, and the bands were visualized by staining with Gel-red for 10 min. (C) MST assay to test the binding capability of purified *P*. *aeruginosa* RplI-His to the indicated RNA (same as A, B) transcribed in vitro. The error bar represents the standard deviation from triplicate assays for each sample.

It has been reported that some ribosomal proteins, such as primary rRNA-binding proteins S1, S7, S8 and L1, interact with mRNAs of their own or others in the operons to inhibit the translation of target genes in *E*. *coli* [19–22]. Since L9 is the primary 23S rRNA-binding protein in the 50S ribosome, we asked if L9 repressed the translation of *exsA* through direct binding to the 24-nt 5’ UTR of *exsA*. To address this, we examined the RplI interaction with the 5’ UTR of *exsA* by EMSA. The RNA of -24 nt-*exsA* or -12 nt-*exsA* of *P*. *aeruginosa* was transcribed with specific primers (S3 Table) from corresponding PCR products *in vitro* and incubated with the indicated amounts of purified C-terminal 6×His tagged RplI of *P*. *aeruginosa*. The -24 nt/12 nt -*exsA* probe is comprised of the native 24-nt/12-nt 5’ UTR and 143 nt downstream of the start codon for *exsA*. As shown in Fig 7A and 7B, RplI of *P*. *aeruginosa* retarded the migration of RNA containing partial *exsA* with its 24 nt 5’ UTR but not that with the 12 nt 5’ UTR, indicating a direct interaction with the 24 nt 5’ UTR of *exsA*.

To further confirm the direct interaction, a microscale thermophoresis assay (MST) was carried out using NT-647-NHS labeled RplI as an aptamer probe. As the results shown in Fig 7C, binding between RplI and the -24 nt-*exsA* (same RNA probe as in EMSA) was detected with a *K*_d_ of 529 ± 272 nM, while interaction between the -12 nt-*exsA* (same RNA probe as in EMSA) and RplI was undetectable. These results further confirmed that RplI interacts with the 24 nt 5’ UTR of *exsA* directly.

### RplI inhibits *exsA* translation via its 24 nt 5’ UTR

**Fig 8:**
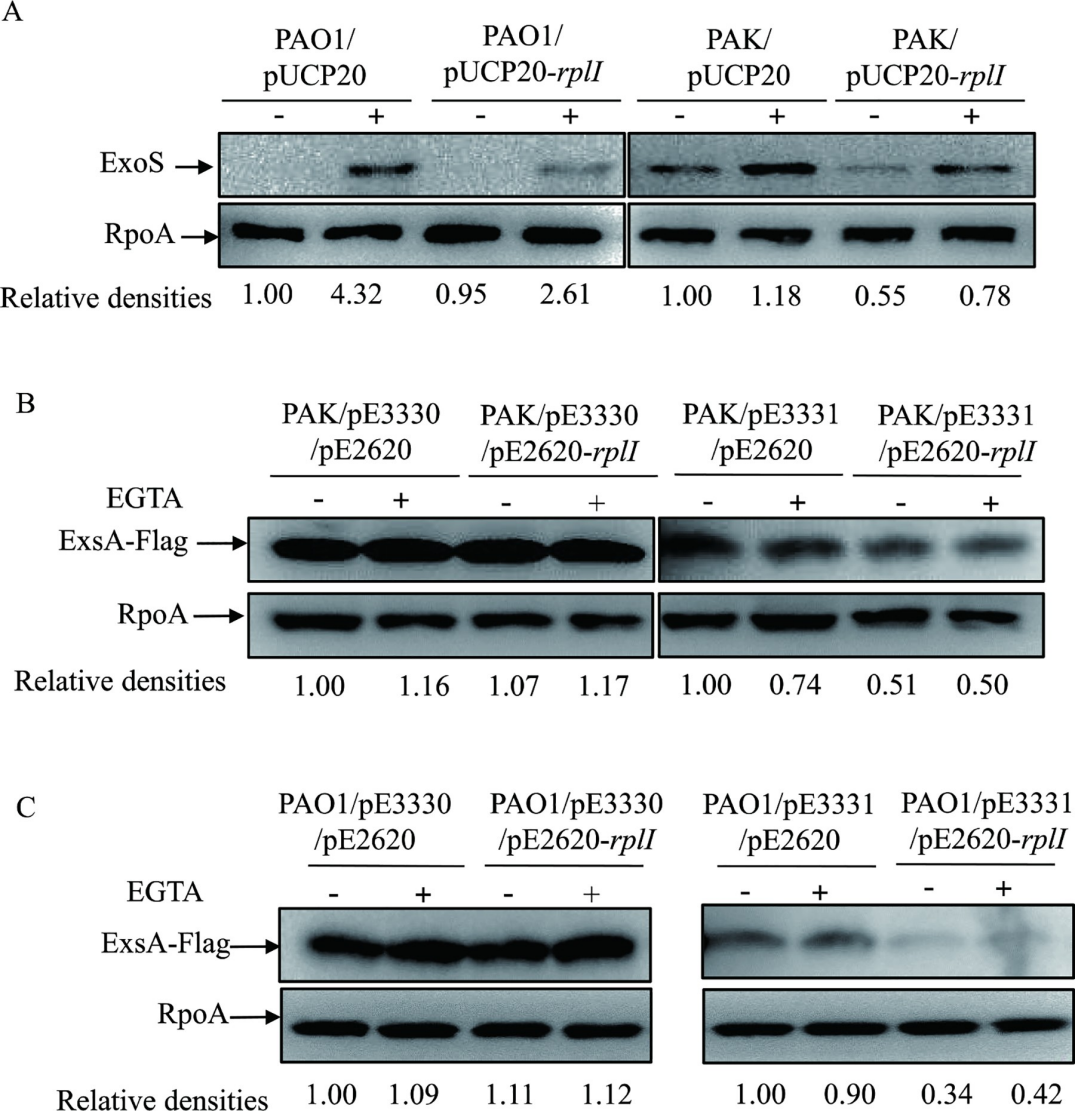
RplI represses the expression of ExsA and ExoS in PAK and PAO1 strains. (A) Expression of ExoS in another PAO1 laboratory strain and PAK containing empty vector pUCP20 or pUCP20-*rplI*. (B and C) Expression of ExsA-Flag in PAK (B) and PAO1 (C) containing pE3330 or pE3331 with empty vector pE2620 or pE2620-*rplI*. Bacteria cultured overnight were diluted 1:50 into fresh LB medium, grown to an OD_600_ of 1.0 with (+) or without (-) 5 mM EGTA and collected by centrifugation. Proteins from equivalent bacterial cells were separated by SDS-PAGE gels and probed with an antibody against ExoS/Flag or an anti-RpoA antibody. The density of each band was determined with Image J. Relative densities represent the density of ExoS or ExsA-Flag/density of RpoA with the respective first lane as 1. The data shown represent the results from three independent experiments.

If RplI represses *exsA* translation by binding to its 24 nt 5’ UTR, overexpression of *rplI* in wild type *P*. *aeruginosa* strains should decrease the expression of ExoS, as well as the translation of *exsA* with its 24 nt 5’ UTR, but not with its 12 nt 5’ UTR. To test these, RplI was overexpressed in the T3SS-proficient *P*. *aeruginosa* laboratory strains PAK and another PAO1 strain. Overexpression of *rplI* indeed decreased the expression of ExoS in both PAK and PAO1 (Figs 8A and S3A). Furthermore, pE2620-*rplI* was introduced into the PAO1 or PAK strain containing pE3331 (24 nt 5’ UTR-*exsA*) or pE3330 (12 nt 5’ UTR-*exsA*). Consistent with the expression of ExoS, overexpression of *rplI* decreased the ExsA-Flag amounts in the presence of its 24 nt 5’ UTR in both PAK and PAO1 strains. However, overexpression of *rplI* in PAK or PAO1 had no effect on the expression of ExsA-Flag in the presence of its 12 nt 5’ UTR (Figs 8B, 8C, S3B and S3C). These results further confirmed that RplI represses the expression of T3SS in *P*. *aeruginosa*.

## Discussion

The bacterial *rplI* gene encodes ribosomal large subunit (50S) protein L9 which is a primary 23S rRNA binding protein with N- and C-terminal globular domains, both containing a predicted RNA binding site connected by an exposed α-helix [23]. It was speculated that L9 functions to serve as a molecular strut, likely playing an architectural role in ribosome assembly and/or maintaining the catalytically active conformation of ribosomal RNA in the large ribosomal subunit by binding and positioning two regions of the 23S rRNA [24,25]. In addition to its role as a ribosomal 23S rRNA-binding protein, L9 has been reported to play an important role in reading frame maintenance in *Salmonella enterica* serovar Typhimurium [13], ribosomal “hopping” over a 50 nucleotide region within the mRNA of bacteriophage T4 gene 60 in *E*. *coli*, and response to starvation stress in *E*. *coli* [26]. Although bacterial L9 possesses a highly conserved secondary and tertiary structure and contains several invariant amino acids, L9 is not essential, and *rplI* deletion strains seem to grow fine under normal conditions in *S*. *enterica* [13].

In this study, we identified that L9 interacts with the 5’ UTR of *exsA* and serves as a translational repressor, suppressing the T3SS in *P*. *aeruginosa*. Small RNA regulators (sRNAs) often sequester the ribosome binding site (RBS) of target genes via intramolecular base-pairing with the 5’ UTR of target mRNAs to downregulate translation initiation. Sr0161 was recently reported to directly target the *exsA* leader region through imperfect base pairing to repress the T3SS [9,10]. It has also been demonstrated that Sr0161 inhibits ExsA synthesis at the posttranscriptional level [10]. As the RplI mediated repression of ExsA translation also involves the *exsA* 5’ untranslated region, a possible model to account for the inhibitory activity of RplI is that imperfect base pairing of Sr0161with the *exsA* leader region prevents the loading of wild-type ribosomes, but not RplI-deficient ribosomes. However, our finding that overexpression or deletion of Sr0161 has the same influence on the expression of ExsA in both mPAO1 and the Δ*rplI* mutant (Fig 6) suggests independence of RplI from Sr0161-mediated regulation of the T3SS in *P*. *aeruginosa*. While both RplI and Sr0161 control ExsA translation via the 5’ UTR of *exsA*, RplI works independent of Sr0161, indicating the possibility that they may function as T3SS repressors but respond to different environmental cues.

There are several regulators that have been shown to regulate the expression of T3SS genes at the posttranscriptional level. *Intile*. *et*. *al*. reported that the RNA helicase DeaD directly stimulates *exsA* translation to promote the expression of T3SS in *P*. *aeruginosa* [8]. DeaD likely functions by relaxing an inhibitory mRNA secondary structure in the *exsA* 5’ untranslated region to enhance ribosomal recruitment [8]. Our previous study showed that the *suhB* mutation is linked to a defective translation of the ExsA [17]. RsmA, a CsrA family RNA binding protein, exerts a positive effect on the synthesis of ExsA through an unknown mechanism [11]. In addition, Sr0161 directly targets the leader region through base pairing and inhibits ExsA synthesis at the posttranscriptional level [9,10]. Recently, a CspA family protein (CspC) was demonstrated to control the translation of *exsA* through its 74 nt 5′ UTR [27]. Here, we further identified the ribosomal large subunit protein RplI to inhibit ExsA synthesis at the translational level.

Genes coding for ribosomal proteins usually form operons in bacteria. It has been demonstrated that numerous ribosomal proteins in *E*. *coli* autogenously repress the translation of their own mRNAs or others in the operons by direct binding to their respective mRNAs [28]. These proteins include both small subunit ribosomal proteins, such as S4 and S7 [29], and those of large subunit ribosomal proteins, such as L1, L10 and L7/L12 [30,31]. Such a feedback regulation is derived from the capacities of these ribosomal proteins to function not only as primary rRNA binding components but also as highly specific repressors for their own mRNAs when they are produced in excess over the rRNA available for the ribosome assembly [28–32]. In *E*. *coli*, the translational repression by ribosomal proteins may occur either through competing with and hampering ribosome binding to mRNA or by entrapment of the ribosome in its nonproductive complex [28]. Since a much higher amount of ribosome association with the *exsA* mRNA was detected in the Δ*rplI* mutant, it is possible that RplI interacts with the 5’ UTR and prevents ribosome binding, thus inhibiting the initiation of *exsA* translation. To the best of our knowledge, this is the first report on ribosomal protein inhibiting mRNA translation beyond its own mRNA via direct binding.

Previous work has shown that a 778 nucleotide RNA fragment ranging from nucleotide 1999 to 2776 of the 23 S rRNA contains two RNA binding sites of ribosomal protein L9 in *E*. *coli* [24]. With the toeprinting assay, they also revealed that the N- and C-terminal domains of L9 respectively interact with nucleotides just 5’ to nucleotides 2231 and 2179 of the 23S rRNA [24]. The interaction between RplI and the mRNA fragment of *exsA* in the presence of its 24-nt 5’ untranslated region was detected, but not in the presence of the 12-nt 5’ UTR, which suggested a specific interaction between the region from the 24 nt to 12 nt 5’ UTR. It is possible that RplI binds to the 24 nt 5’ UTR and affects the binding of ribosomes to *exsA* mRNA. However, no obvious primary sequence homolog was found at -24 nt-*exsA* when compared to 23S rRNA. Since the mRNA secondary structure linking sequences upstream and downstream of the RBS of *rpsM* was important for the S4 mediated translational repression [33,34], it is possible that the stem-loop structure of 24 nt upstream of the coding sequence of *exsA* (S4 Fig predicted with Mfold) is responsible for its binding and repression by the L9.

Taken together, our study showed that RplI binds to the 5’ UTR of and inhibits *exsA* translation to repress expression of the T3SS in *P*. *aeruginosa*. Further studies are required to understand the global regulatory role of the L9 and their detailed regulatory mechanisms in *P*. *aeruginosa*.

## Materials and methods

### Bacterial strains and plasmids

Bacteria and plasmids used in this study are listed in S2 Table. All strains were cultured in LB medium (10 g/L tryptone, 5 g/L yeast extract and 5 g/L NaCl) or on L-agar plates at 37°C. When needed, antibiotics were used at the following concentrations (μg/mL): for *E*. *coli*, ampicillin 100, gentamicine 10, tetracycline 10; for *P*. *aeruginosa*, carbenicillin 150, gentamicin 100, tetracycline 50. When needed, IPTG (isopropyl β-D-1-thiogalactopyranoside) at the indicated concentrations was added into the culture medium. Primers used to generate constructs and in real-time qPCR are listed in S3 Table.

For complementation of the *rplI* gene, the open reading frame of *rplI* and its putative SD region was PCR amplified with mPAO1 chromosomal DNA as the template. The PCR product was ligated into the *Bam*HI-*Hin*dIII sites of pUCP20 [35], resulting in pUCP20-*rplI*. pE2620-Sr0161 was constructed with similar manipulation. To generate pE2620, a Gm^r^ fragment was amplified from pUC18T-miniTn7T [36] with primers pE2620F and pE2620R and cloned into the *Pvu*I site of pMMB67EH (ATCC). The *rplI* deletion construct was made by cloning 1034 bp upstream and 1075 bp downstream fragments of the *rplI* gene into the *Sac*I-*Hin*dIII sites of plasmid pEX18Tc [37]. pEX18-Sr0161 were constructed with similar manipulation. To generate the -12 bp-*exsA*-Flag construct, the ORF of *exsA* (without a stop codon) and its 12 bp upstream fragment was cloned into the *Hin*dIII-*Kpn*I sites of RBS-free pFlag-CTC, in which RBS was removed by PCR amplification with primers pFlag-CTCF and pFalg-CTCR (S3 Table) from pFlag-CTC (Sigma) followed by cloning into its *Bam*HI-*Hin*dIII sites. The resulting construct was inserted into the *Bam*HI site of pDN19 [38], generating pE3286. pE269, pE268, pE267 and pE3308 were constructed with similar manipulations. To construct pE3330 and pE3331, -12 bp-*exsA*-Flag and -24 bp-*exsA*-flag with the *tac* promoter of pFlag-CTC were PCR amplified with pE3286 and pE267 as templates using primers P_*tac*_F and P_*ter*_R (S3 Table), and then cloned into the *Sac*I-*Bam*HI sites of pE1553 [16]. pE1553 was used in a previous study [16] and generated by removing the promoter of pUCP20 [35] via point mutation using PCR with primers pE1553F and pE1553R (S3 Table) followed by digestion with *Eco*RI and self-ligation.

### Western blot assay

Bacteria cultured overnight were diluted 1:50 into fresh LB medium and grown to an OD_600_ of 1.0 with or without 5 mM EGTA. After separation by centrifugation at 13, 000 × g for 2 min, supernatant and/or pellet samples from an equal number of cells were separated on a 12% sodium dodecyl sulfate-polyacrylamide gel (SDS-PAGE), transferred onto a PVDF (polyvinylidene difluoride) membrane (Millipore), and probed with a rabbit polyclonal antibody against ExoS or mouse monoclonal antibody against RpoA, RpoB (BioLegend) and Flag (Sigma). The RNA polymerase alpha/beta subunit (RpoA/RpoB) was used as a loading control. Signals were detected with the ECL-plus kit (Millipore).

### Cytotoxicity assay

Bacterial cytotoxicity was determined by examining the detachment of HeLa cells after *P*. *aeruginosa* infection as previously described [14]. 1.4 × 10^5^ HeLa cells were seeded into each well of a 24-well plate and cultured in DMEM (Dulbecco’s modified Eagle’s medium, Corning, USA) containing 10% fetal bovine serum (FBS, Gibco) and 1% penicillin/streptomycin (Gibco, USA) at 37°C with 5% CO2 the night before infection. Three hours before infection, cells were washed twice with phosphate-buffered saline (PBS) and further cultured in DMEM with 10% FBS. Before infection, log phase bacteria grown in LB medium were collected, washed twice with PBS, and resuspended in DMEM with 10% FBS. Then HeLa cells were infected with log phase bacteria at a multiplicity of infection (MOI) of 50 for 3 hours. After that, the culture medium was removed, and the remaining attached cells were washed twice with PBS and stained with 0.1% crystal violet for 15 min at 37°C. Then, each well was washed twice with 1 mL distilled water. The stained crystal violet was dissolved in 95% ethanol and measured at a wavelength of 590 nm.

### RNA isolation and real-time qPCR

RNA purification and real-time qPCR were carried out following the manufacturer’s instructions with minor modifications as described below. Bacteria cultured overnight were diluted 1:50 into fresh LB medium and grown to an OD_600_ of 1.0 at 37°C with or without 5 mM EGTA. Total RNA was extracted using an RNAprep Pure Cell/Bacteria Kit (Tiangen Biotech, Beijing, China). cDNA was synthesized using random primers and PrimeScript Reverse Transcriptase (TaKaRa, Dalian, China). Real-time qPCR was performed with the indicated primers (shown in S3 Table) and SYBR Premix ExTaq II (TaKaRa, Dalian, China). *rpsL*, a 30S ribosomal protein encoding gene, was used as an internal control.

### mRNA stability assay

mRNA stability assay was performed as described before with minor modifications [39]. *P*. *aeruginosa* strains cultured overnight were diluted 1:50 into fresh LB medium containing 5 mM EGTA or not, grown to an OD_600_ of 1.0, and treated with 200 μg/mL rifampicin. Equal numbers of bacterial cells were collected at each indicated time point. Then each sample was mixed with an equivalent number of *gfp*-expressing *E*. *coli* cells. Total RNA was isolated, and the mRNA levels of *rpsL*, PA1805 (the peptidyl-prolyl cis-trans isomerase D encoding gene) and *exsA* were examined by real-time qPCR with *gfp* as an internal control.

### Detection of *exsA* mRNA associated with ribosome

The amount of ribosome-associated *exsA* mRNA was determined by a modified RNA-binding protein immunoprecipitation procedure followed by real-time qPCR [18,40,41]. Flag-tagged *exsA* coding sequence with its 24 bp upstream region followed by transcription terminators T0 and T1 was PCR amplified from pE267 plasmid and then cloned into pE1553, resulting in construct pE3331. pE3330 containing -12 bp-*exsA*-Flag-T0T1 was generated with the same procedure. Wild-type mPAO1 and its Δ*rplI* mutant containing pE2620-*rplL*-His and pE3331 or pE3330 were grown to an OD_600_ of 1.0 and collected by centrifugation. Ribosome was isolated by affinity chromatography as described previously with modifications [42]. The collected bacteria were suspended in lysis buffer (150 mM NaCl, 20 mM Tris-HCl, 10 mM imidazole, 3 mM β-mercaptoethanol, 0.5% NP-40, pH 8.0) and lysed by sonication. Cell debris was removed by centrifugation, and the supernatants were incubated with Ni-NTA agarose beads at 4°C for 1 h. After that, the beads were washed five times with lysis buffer, followed by RNA purification with an RNAprep Pure Cell/Bacteria kit (Tiangen Biotech, China). The amount of *exsA*-Flag mRNA was determined by real-time qPCR with specific primers (q*exsA*FlagF and q*exsA*FlagR in S3 Table) and 16S ribosomal RNA (PA0668.1) as the internal control for normalization.

### Expression and purification of proteins from *E*. *coli*

The *rplI* coding region was amplified by PCR using mPAO1 chromosome DNA as the template with specific primers (S3 Table). The gene was cloned into the *Nco*I-*Xho*I sites of plasmid pET28a, resulting in *rplI* translational fusion with the His tag at the C-terminus. *E*. *coli* strain BL21 (DE3) carrying the plasmid pET28a-*rplI* was cultured at 37°C to an OD_600_ of 0.4 to 0.6, and expression of His-tagged RplI was induced by the addition of IPTG at a final concentration of 100 μM for 16~18 h at 16°C. Bacteria from 100 mL culture were collected by centrifugation at 4,000 × g for 20 min, resuspended in 10 mL lysis buffer (46.6 mM Na_2_HPO_4_, 3.4 mM NaH_2_PO_4_, 0.3 M NaCl, 8 M urea, pH 8.0) and lysed by sonication on ice. The lysate was centrifuged at 12,000 × g for 20 min at room temperature. Then, the supernatant was mixed with Ni-NTA agarose (Qiagen) and incubated at room temperature for 1 h. After that, the lysate-resin mixture was loaded into an empty column and washed twice with lysis buffer containing 50 mM imidazole. The RplI-His protein was finally eluted with l mL lysis buffer containing 300 mM imidazole. To remove urea and imidazole, proteins were extensively dialyzed in PBS with various concentrations of urea (4, 2, and 1 M) and finally in PBS.

### *In vitro* transcription and RNA gel mobility shift assay

The -24 nt-*exsA*/ -12 nt-*exsA* RNA was synthesized using the Riboprobe System-T7 (Promega) from PCR product amplified from mPAO1 chromosomal DNA with the specific primers (listed in S3 Table) according to the manufacturer’s instructions. The RNA was purified by isopropanol precipitation, heated at 90°C for 10 min and then refolded by cooling naturally at room temperature for 30 min. One microgram of the purified RNA was mixed with the indicated amounts of purified RplI-His in binding buffer (10 mM Tris-HCl, pH 7.5, 5 mM MgCl_2_, 50 mM KCl, 10% glycerol, 1 U recombinant RNase inhibitor [TaKaRa]) and incubated for 30 min on ice. Fifteen microliters of each sample was loaded onto a nondenaturing 8% polyacrylamide gel. Electrophoresis was performed at 100 V for 145 min in 1 × TBE buffer (Tris-borate-EDTA: 89 mM Tris, 89 mM boric acid, 2 mM EDTA, pH 8.3) on ice. After that, the gel was stained with Gel-red (Biotium) in 1 × TBE for 10 min and the RNA bands were visualized in a ChemiDoc XRS+ molecular imager (Biorad, CA, USA). To avoid RNase contamination, all buffers were prepared with DEPC-treated water.

### Microscale thermophoresis measurements

The purified RplI-His was labeled with the RED fluorescent dye NT-647-NHS (NanoTemper Technologies, Munich, Germany) following the manufacturer’s instructions. 200 nM labeled RplI-His was incubated with a 2-fold dilution series of unlabeled indicated RNA (with final concentrations ranging from 15.9 μM to 0.485 nM) in a twenty microliter system on ice for at least 30 min [43]. Following incubation, the samples were loaded into standard treated silicon capillaries (Monolith NT.115 series capillaries; catalog no. MO-K025). The measurements were carried out using a Monolith NT.115 instrument (NanoTemper Technologies GmbH) at room temperature using a 20% light-emitting diode (LED) and 60% MST power. The dissociation constants (*K*_*d*_) were calculated as described previously [44]. Data analyses were performed using MO affinity analysis software (NanoTemper Technologies). The whole procedure was carried out in triplicate for each sample.

### Other methods

Tn5 mutagenesis was conducted as previously described [45,46]. Chromosomal gene deletion was carried out by homologous recombination as previously described [37]. β-galactosidase activity was measured to determine the indicated promoter transcriptional activity as described before [47].

## Supporting information

S1 Table Tn5 insertion mutants with increased T3SS expression. (DOCX)

S2 Table Strains and plasmids used in this study. (DOCX)

S3 Table Primers used in this study. (DOC)

S1 Fig Growth curve of mPAO1 and Δ*rplI* in LB medium (A), relative mRNA levels of *pcrV* and *pscF* in mPAO1 and Δ*rplI* strains (B), secretion and expression of ExoS in PAO1/pUCP20, PAO1Δ*rplI*/pUCP20 and PAO1Δ*rplI*/pUCP20*-rplI* (C). (B) Total RNA was isolated under T3SS inducing (+) and non-inducing (-) conditions with 5 mM EGTA, and the relative mRNA levels of *pcrV* and *pscF* were determined by real-time qPCR using *rpsL* as the internal control. ns, not significant, *P < 0.05, ***P < 0.001 by Student’s *t* test. (C) Bacteria were cultured to an OD_600_ of 1.0 in LB with (+) or without (-) 5 mM EGTA. Proteins in supernatants (S) or pellets (P) from equivalent bacterial cells were separated by 12% SDS-PAGE gels and probed with an antibody against ExoS or RpoA.
(TIF)

S2 Fig Bar diagrams of the relative densities of Western blots in Figs 2B (S2A and S2B Fig) and 3E (S2C Fig). The relative grayscale represents the density of the sample/density of the loading control with the respective first lane as 1. ** *P* < 0.01, ***P< 0.001, ****P < 0.0001, by Student’s *t* test. The data shown represent the results from three independent experiments.
(TIF)

S3 Fig Bar diagrams of the relative densities of Western blots in Fig 8A (S3A Fig), 8B (S3B Fig) and 8C (S3C Fig). The relative grayscale represents the density of the sample/density of the loading control with the respective first lane as 1. ns, not significant, ***P< 0.001, ****P < 0.0001, by Student’s *t* test. The data shown represent the results from three independent experiments.
(TIF)

S4 Fig Secondary structure of 24 nt 5’ untranslated region of *exsA*, predicted by Mfold. The region from -24 nt to -12 nt was boxed.
(TIF)
