# Supplementary material for: RplI interacts with 5’ UTR of exsA to repress its translation and type III secretion system in Pseudomonas aeruginosa
Source: PLoS Pathog. 2022 Jan 5;18(1):e1010170. doi: 10.1371/journal.ppat.1010170 (PMC8730436; doi:10.1371/journal.ppat.1010170)
Supplement: S1 Table — (DOCX) [file ppat.1010170.s001.docx]

**S1 Table.** Tn5 insertion mutants with increased T3SS expression

| **Gene locus/**  **Intergenic region** | **# of mutants recovered** | **Independent Tn5 insertion sites** | **Gene product** |
| --- | --- | --- | --- |
| Gene locus |  |  |  |
| PA2493 | 8 | 2 | Resistance-Nodulation-Cell Division (RND) multidrug efflux membrane fusion protein MexE precursor |
| PA2689 | 3 | 1 | Esterase (PfeE) |
| PA2800 | 4 | 1 | VacJ |
| PA3159 | 4 | 2 | UDP-N-acetyl-d-glucosamine 6-Dehydrogenase |
| PA4932 | 2 | 2 | 50S ribosomal protein L9 |
| PA5268 | 4 | 2 | Magnesium/cobalt transport protein |
| intergenic region |  |  |  |
| PA4743-PA4744 | 6 | 2 | PA4743: ribosome-binding factor A  PA4744: Translation initiation factor IF-2 |
| PA4744-PA4745 | 3 | 1 | PA4744: Translation initiation factor IF-2  PA4745: N utilization substance protein A |
|  |  |  |  |
