# Supplementary material for: RplI interacts with 5’ UTR of exsA to repress its translation and type III secretion system in Pseudomonas aeruginosa
Source: PLoS Pathog. 2022 Jan 5;18(1):e1010170. doi: 10.1371/journal.ppat.1010170 (PMC8730436; doi:10.1371/journal.ppat.1010170)
Supplement: S2 Table — (DOCX) [file ppat.1010170.s002.docx]

**S2 Table.** Bacterial strains and plasmids used in this study.

| Strains or plasmids | Description | Source or reference |
| --- | --- | --- |
| **strains** |  |  |
| DH5α | F^̶^ ϕ 80d*lacZ*∆M15 *endA1 recA1 hsdR17*(r_K_^̶^ m_K_^+^) *supE44 thi-1 relA1* ∆(*lacZYA-argF*)*U169 gyrA96 deoR* | TransGen |
| S17-1 | RP4-2 Tc::Mu Km::Tn*7* Tp^r^ Sm^r^ Pro Res^̶^ Mod^+^ | Stratagene |
| BL21 (DE3) | F^-^ *ompT* *hsdSB (rB-, mB-) gal dcm* (DE3) | invitrogen |
|  |  |  |
| BW20767/pRL27 | RP4-2-Tc::Mu-1 Kan::Tn7 integrant *leu-63*::IS10 *recA1 zbf-5* *creB510 hsdR17* *endA1 thi uidA* (∆MluI::*pir*)/pRL27 | [[1](#_ENREF_1)] |
| mPAO1 | Wild type *P. aeruginosa* strain | [[2](#_ENREF_2)] |
| PAK | Wild type *P. aeruginosa* strain | [[2](#_ENREF_2)] |
| PAO1 | Wild type *P. aeruginosa* strain | [[3](#_ENREF_3)] |
| Δ*rplI* | mPAO1 with *rplI* gene deleted | This study |
| Δ*rplI*/pUCP20*-rplI* | Overexpression of *rplI* with pUCP20 in Δ*rplI* mutant | This study |
| ΔSr0161 | mPAO1 with Sr0161 gene deleted | This study |
| Δ*rplI*ΔSr0161 | mPAO1 with both *rplI* and Sr0161 deleted | This study |
| PAO1Δ*rplI* | PAO1 with *rplI* gene deleted | This study |
| **Plasmids** |  |  |
| pUCP20 | Shuttle vector between *E. coli* and *P. aeruginosa*; Amp^r^ | [[4](#_ENREF_4)] |
| pE2620 | pMMB67EH-Gm, Shuttle vector between *E. coli* and *P. aeruginosa*; Gm^r^ | [[5](#_ENREF_5)] |
| pE1553 | Promoterless pUCP20, pUCP20 with promoter removed; Amp^r^ | [[6](#_ENREF_6)] |
| pET28a | Expression vector, Kan^r^ | Novagen |
| pET28a-*rplI* | *rplI* gene from mPAO1 in pET28a driven by T7 promoter, Kan^r^ | This study |
| pEX18Tc | Gene knockout vector; Tc^r^ | [[7](#_ENREF_7)] |
| pUCP20-*rplI* | *rplI* gene from mPAO1 in pUCP20; Amp^r^ | This study |
| pE2620-Sr0161 | Sr0161 gene from mPAO1 in pMMB67EH; Gm^r^ | This study |
| pE2620-*rplI* | *rplI* gene from mPAO1 in pMMB67EH driven by *tac* promoter; Gm^r^ | This study |
| pE2620-*rplL*-His | *rplL* gene from mPAO1 in pMMB67EH; Gm^r^ | This study |
| pEX18-*rplI* | *rplI* gene deletion on pEX18Tc; Tc^r^ | This study |
| pEX18-Sr0161 | Sr0161 gene deletion on pEX18Tc; Tc^r^ | This study |
| pE643 | *exsA* gene with Flag tagged on pUC18T-mini-Tn7T driven by *exsC* promoter; Gm^r^ | This study |
| pE705 | *exsCEBA* gene with *exsA*-Flag tagged on pUC18T-mini-Tn7T driven by *exsC* promoter; Gm^r^ | [[8](#_ENREF_8)] |
| pE2120 | *exsA-*flag with its own promoter in pE1553 | [[6](#_ENREF_6)] |
| pE117 | *exsA* ORF and 225bp upstream fragment cloned into pFlag-CTC-pDN19 driven by tac promoter; Tc^r^, Amp^r^ | [[9](#_ENREF_9)] |
| pE269 | *exsA* ORF and 120bp upstream fragment cloned into pFlag-CTC-pDN19 driven by tac promoter; Tc^r^, Amp^r^ | This study |
| pE268 | *exsA* ORF and 74bp upstream fragment cloned into pFlag-CTC-pDN19 driven by tac promoter; Tc^r^, Amp^r^ | This study |
| pE267 | *exsA* ORF and 24bp upstream fragment cloned into pFlag-CTC-pDN19 driven by tac promoter; Tc^r^, Amp^r^ | This study |
| pE3286 | *exsA* ORF and 12bp upstream fragment cloned into pFlag-CTC-pDN19 driven by tac promoter; Tc^r^, Amp^r^ | This study |
| pE3308 | *gfp* ORF and 24bp upstream fragment of *exsA* ORF cloned into pFlag-CTC-pDN19 driven by tac promoter; Tc^r^, Amp^r^ | This study |
| pE3330 | -12bp*exsA-*flag cloned into pE1553 driven by tac promoter; Amp^r^ | This study |
| pE3331 | -24bp*exsA-*flag cloned into pE1553 driven by tac promoter; Amp^r^ | This study |
| P*_exsA_*-*lacZ* | *exsA* promoter fused to promoterless *lac*Z on pDN19*lac*ZΩ; Sp^r^, Sm^r^, Tc^r^ | [[6](#_ENREF_6)] |
| P*_exsC_*-*lacZ* | *exsC* promoter fused to promoterless *lac*Z on pDN19*lac*ZΩ; Sp^r^, Sm^r^, Tc^r^ | [[6](#_ENREF_6)] |

1. Larsen, R.A., et al., *Genetic analysis of pigment biosynthesis in Xanthobacter autotrophicus Py2 using a new, highly efficient transposon mutagenesis system that is functional in a wide variety of bacteria.* Arch Microbiol, 2002. **178**(3): p. 193-201.

2. Jin, Y., et al., *MexT regulates the type III secretion system through MexS and PtrC in Pseudomonas aeruginosa.* J Bacteriol, 2011. **193**(2): p. 399-410.

3. Liu, C., et al., *Construction of a Protective Vaccine Against Lipopolysaccharide-Heterologous Pseudomonas aeruginosa Strains Based on Expression Profiling of Outer Membrane Proteins During Infection.* Front Immunol, 2018. **9**: p. 1737.

4. West, S.E., et al., *Construction of improved Escherichia-Pseudomonas shuttle vectors derived from pUC18/19 and sequence of the region required for their replication in Pseudomonas aeruginosa.* Gene, 1994. **148**(1): p. 81-6.

5. Long, Y., et al., *Identification of novel genes that promote persister formation by repressing transcription and cell division in Pseudomonas aeruginosa.* J Antimicrob Chemother, 2019. **74**(9): p. 2575-2587.

6. Deng, X., et al., *Fis Regulates Type III Secretion System by Influencing the Transcription of exsA in Pseudomonas aeruginosa Strain PA14.* Front Microbiol, 2017. **8**: p. 669.

7. Schweizer, H.P., *Allelic exchange in Pseudomonas aeruginosa using novel ColE1-type vectors and a family of cassettes containing a portable oriT and the counter-selectable Bacillus subtilis sacB marker.* Mol Microbiol, 1992. **6**(9): p. 1195-204.

8. Jin, Y., et al., *NrtR Regulates the Type III Secretion System Through cAMP/Vfr Pathway in Pseudomonas aeruginosa.* Front Microbiol, 2019. **10**: p. 85.

9. Li, K., et al., *SuhB is a regulator of multiple virulence genes and essential for pathogenesis of Pseudomonas aeruginosa.* mBio, 2013. **4**(6): p. e00419-13.
