# Supplementary material for: RplI interacts with 5’ UTR of exsA to repress its translation and type III secretion system in Pseudomonas aeruginosa
Source: PLoS Pathog. 2022 Jan 5;18(1):e1010170. doi: 10.1371/journal.ppat.1010170 (PMC8730436; doi:10.1371/journal.ppat.1010170)
Supplement: S3 Table — (DOC) [file ppat.1010170.s003.doc]

**S3 Table.** Primers used in this study.

| Primer^a^ | Sequence 5’-3’^b^ |
| --- | --- |
| *rplI*CF | CGGGATCCAAAAGTTAAGAGGTAAGACTCAAATG |
| *rplI*CR | CCCAAGCTTCTACCGATCGGATCGCTTACTCAGC |
| Sr0161OEF | gcTCTAGAAGCTCAATGGATAATTGTCGCTGGCTG |
| Sr0161OER | cccAAGCTTCGGGCGATCGCTCCCATG |
| *rplI*UF | CCGGCAgagctcCCAAGTACCAGCGTCAGC |
| *rplI*UR | GTTGGCGGATCCTTCCAGCAGGATGACTTC |
| *rplI*DF | cgggatccGCGATCCGATCGGTAGGGCTGGGCG |
| *rplI*DR | cccaagcttAGGTCGCCGTGCTCGCGCACCAGGC |
| Sr0161UF | GCGAGCTCCCAAGTGAACAGGGTGTCATCGG |
| Sr0161UR | cgggatccCTTTAGGCTTTCCACGTCCCGCTC |
| Sr0161DF | cgggatccCGGGTTGCGCCCGGCTGGGCGC |
| Sr0161DR | cccaagcttTGTAGTACTTGGTGAAACTGACCTG |
| *exsA*12F | cccaagcttGGTTCTTATAATATGCAAGGAGCC |
| *exsA*R | ggggtaccGTTATTTTTAGCCCGGCATTCGTCC |
| -24*exsAgfp*F | cccaagcttCGGGAAGTGTTGGGGTTCTTATAATATGAGTAAAGGAGAAGAACTTTTCACATGAGTAAAGGAGAAGAACTTTTCAC |
| -24*exsAgfp*R | ggggtaccTTTGTATAGTTCATCCATGCCATG |
| P*_tac_*F | GCGAGCTCGAGCTGTTGACAATTAATCATCGGC |
| terR | cgggatccATAAAACGAAAGGCCCAGTCTTTCG |
| *rplL*F | gctctagaCCAATACAGGAATTAGAGTCATGGC |
| *rplL*R | cccaagcttttaATGATGATGATGATGATGCTTGAGCTCGACTTTGGCGCCTG |
| *rplI*F（pET28a） | CATGCCATGGGCGAAGTCATCCTGCTGGAAAAAGTCGC |
| *rplI*R（pET28a） | CCGCTCGAGCTCAGCGACGATGATCAGCTTCAGAG |
| -24*exsA*-T7F | ACCGCGAAATTAATACGACTCACTATAGGGGGGAAGTGTTGGGGTTCTTATAATATG |
| -12*exsA*-T7F | ACCGCGAAATTAATACGACTCACTATAGGGGGTTCTTATAATATGCAAGGAGCC |
| *exsA*-T7R | GTGGAATCGATGTCCTGGACGGTCAG |
| 23S rRNA-T7F | ACCGCGAAATTAATACGACTCACTATAGGGAGTCTGCATGGAGCCGACCTTG |
| 23S rRNA-T7R | GTTACTCTTTAGGAGGAGACCGCC |
| pE2620-*rplI*F | CCGGAATTCAAAAGTTAAGAGGTAAGACTCAAATG |
| pE2620-*rplI*R | CGGGATCCTTACTCAGCGACGATGATCAGCTTCAG |
| pFlag-CTCF | CGTTGCGCGAGAAGATTGTG |
| pFlag-CTCR | ATGATAAAGCTTGTGTGAAATTGTTATCCGCT |
| pE1553F | CTCACTGCCCGAATTCCAGTCG |
| pE1553R | CCCGACTGGAATTCGGGCAG |
| pE2620F | CTTACTTCTGACAACGATCGTTGACATAAGCCTGTTCGGTTCG |
| pE2620R | CTCCTTCGGTCCTCCGATCGTTAGGTGGCGGTACTTGGGTCG |
| qPCR primer |  |
| q*exoS*F | GCATATTCAATCGCTTCAG |
| q*exoS*R | CCTCAATCTGTCCCAAAC |
| q*exsA*F | GCTATGTCGTAAGTACCA |
| q*exsA*R | GAAGCCTTGTAGAAACTG |
| q*rpsL*F | CAAAACTGCCCGCAACGT |
| q*rpsL*R | TTTCGGCGTGGTGGTGTAT |
| q*exsA*FlagF | ATTTCACCCAGAGCTATC |
| q*exsA*FlagR | CGTCCTTGTAGTCGACAG |
| q*pscF*F | GGCGCAGATATTCAACCCCA |
| q*pscF*R | GGTCACCGTCGAGTTGATGT |
| q*pcrV*F | CCCACGCTCTATGGCTATGC |
| q*pcrV*R | TTGAGTTCCCCGCTCTGCT |
| qPA1805F | ATATCAGTCTCAATGAAGTC |
| qPA1805R | CATGGATGGATCGAAATC |
| q*gfp*F | ATGCCTGAAGGTTATGTA |
| q*gfp*R | TGTGAGTTATAGTTGTATTCC |
| qPA0668.1F | AAGGTCTTCGGATTGTAA |
| qPA0668.1R | GTGCTTATTCTGTTGGTAA |

a: F, forward; R, reverse; U, upstream of specific gene; C, complement; D, downstream of specific gene; OE, overexpression; q, qPCR; b: The underlines are the sites of restriction enzymes.
