# Supplementary figures and images for: RplI interacts with 5’ UTR of exsA to repress its translation and type III secretion system in Pseudomonas aeruginosa
Source: PLoS Pathog. 2022 Jan 5;18(1):e1010170. doi: 10.1371/journal.ppat.1010170 (PMC8730436; doi:10.1371/journal.ppat.1010170)

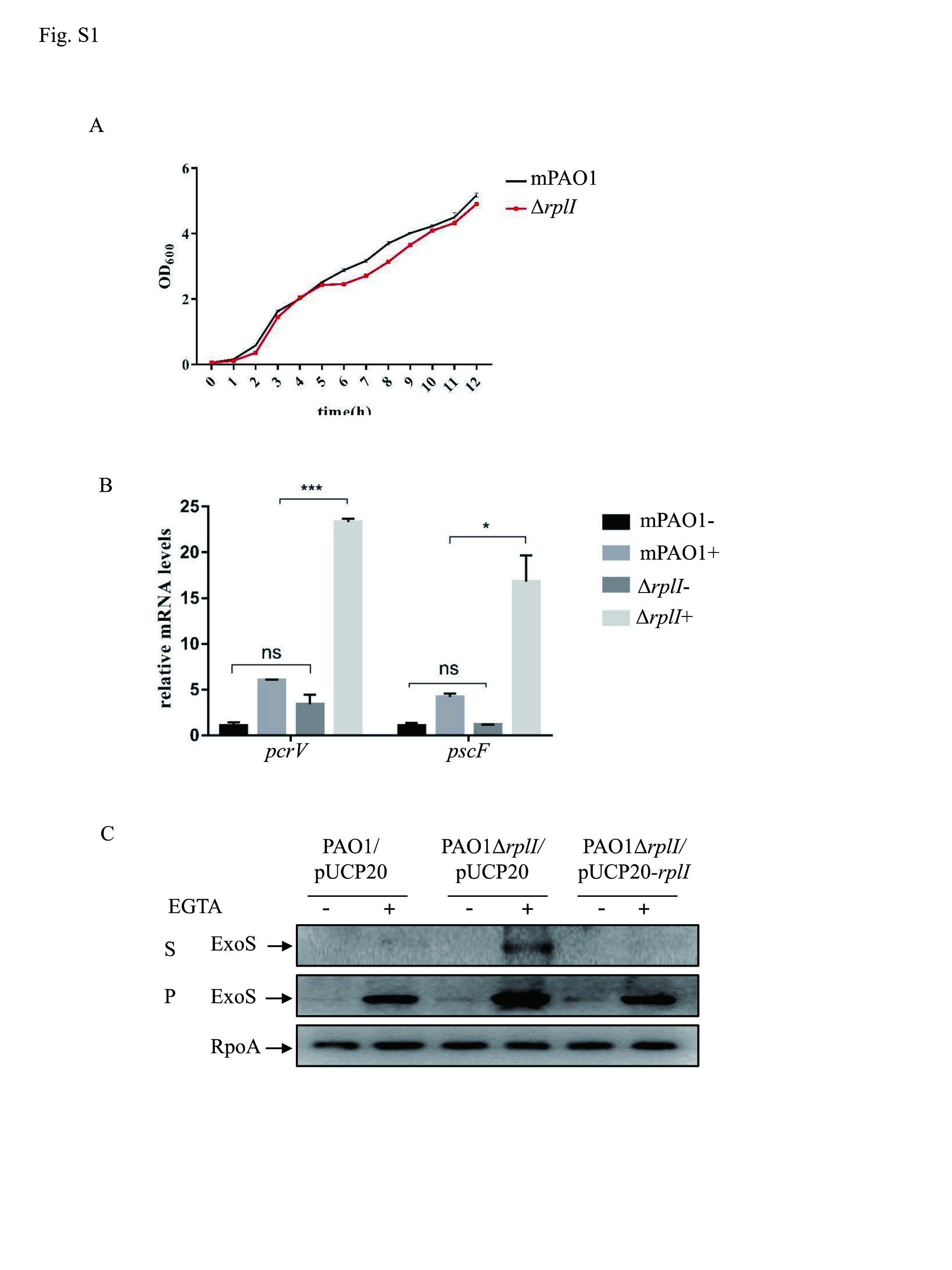

Supplement: S1 Fig — Growth curve of mPAO1 and ΔrplI in LB medium (A), relative mRNA levels of pcrV and pscF in mPAO1 and ΔrplI strains (B), secretion and expression of ExoS in PAO1/pUCP20, PAO1ΔrplI/pUCP20 and PAO1ΔrplI/pUCP20-rplI (C). (B) Total RNA was isolated under T3SS inducing (+) and non-inducing (-) conditions with 5 mM EGTA, and the relative mRNA levels of pcrV and pscF were determined by real-time qPCR using rpsL as the internal control. ns, not significant, *P < 0.05, ***P < 0.001 by Student’s t test. (C) Bacteria were cultured to an OD600 of 1.0 in LB with (+) or without (-) 5 mM EGTA. Proteins in supernatants (S) or pellets (P) from equivalent bacterial cells were separated by 12% SDS-PAGE gels and probed with an antibody against ExoS or RpoA. (TIF) [file ppat.1010170.s004.tif]

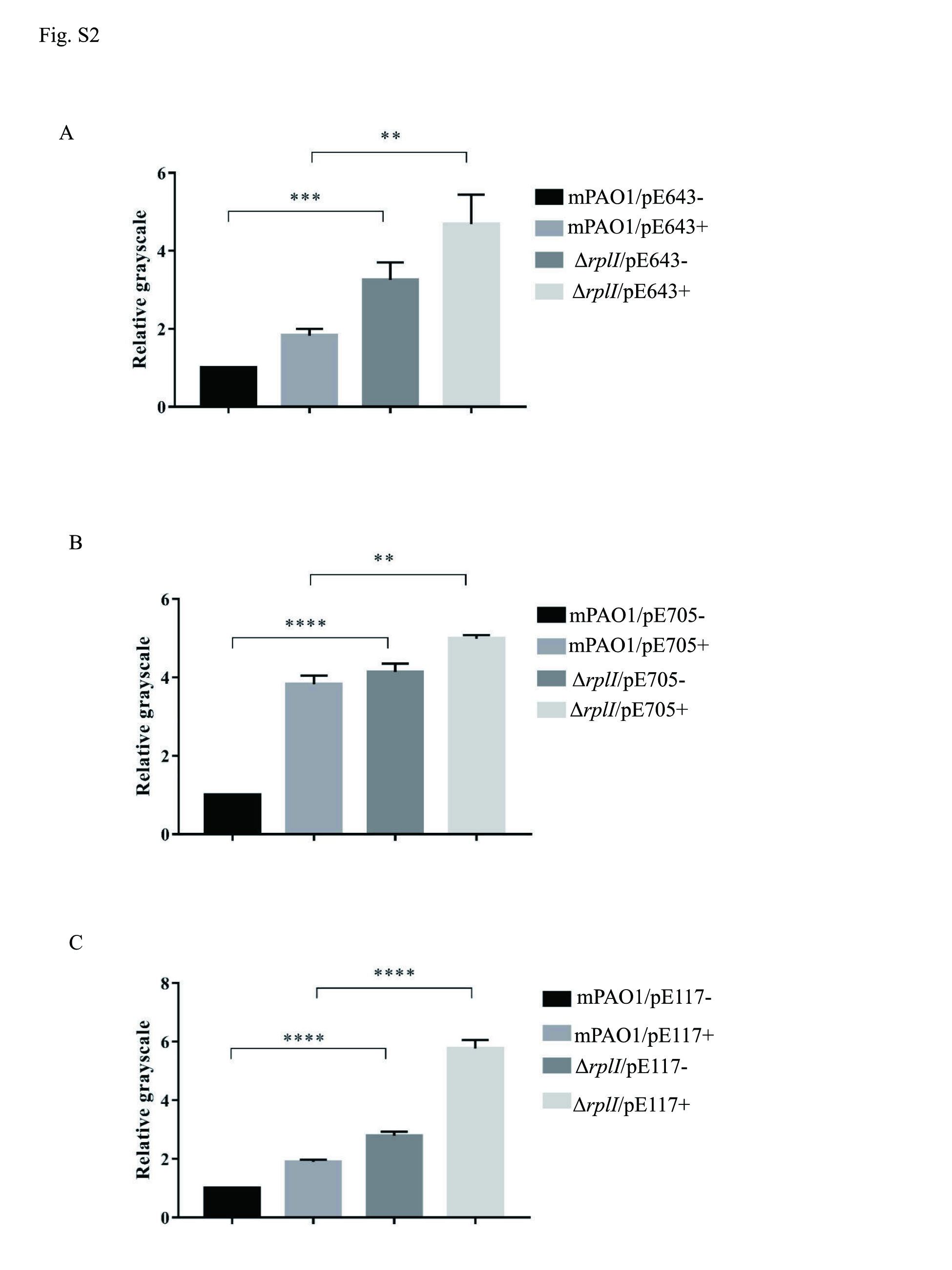

Supplement: S2 Fig — The relative grayscale represents the density of the sample/density of the loading control with the respective first lane as 1. ** P < 0.01, ***P< 0.001, ****P < 0.0001, by Student’s t test. The data shown represent the results from three independent experiments. (TIF) [file ppat.1010170.s005.tif]

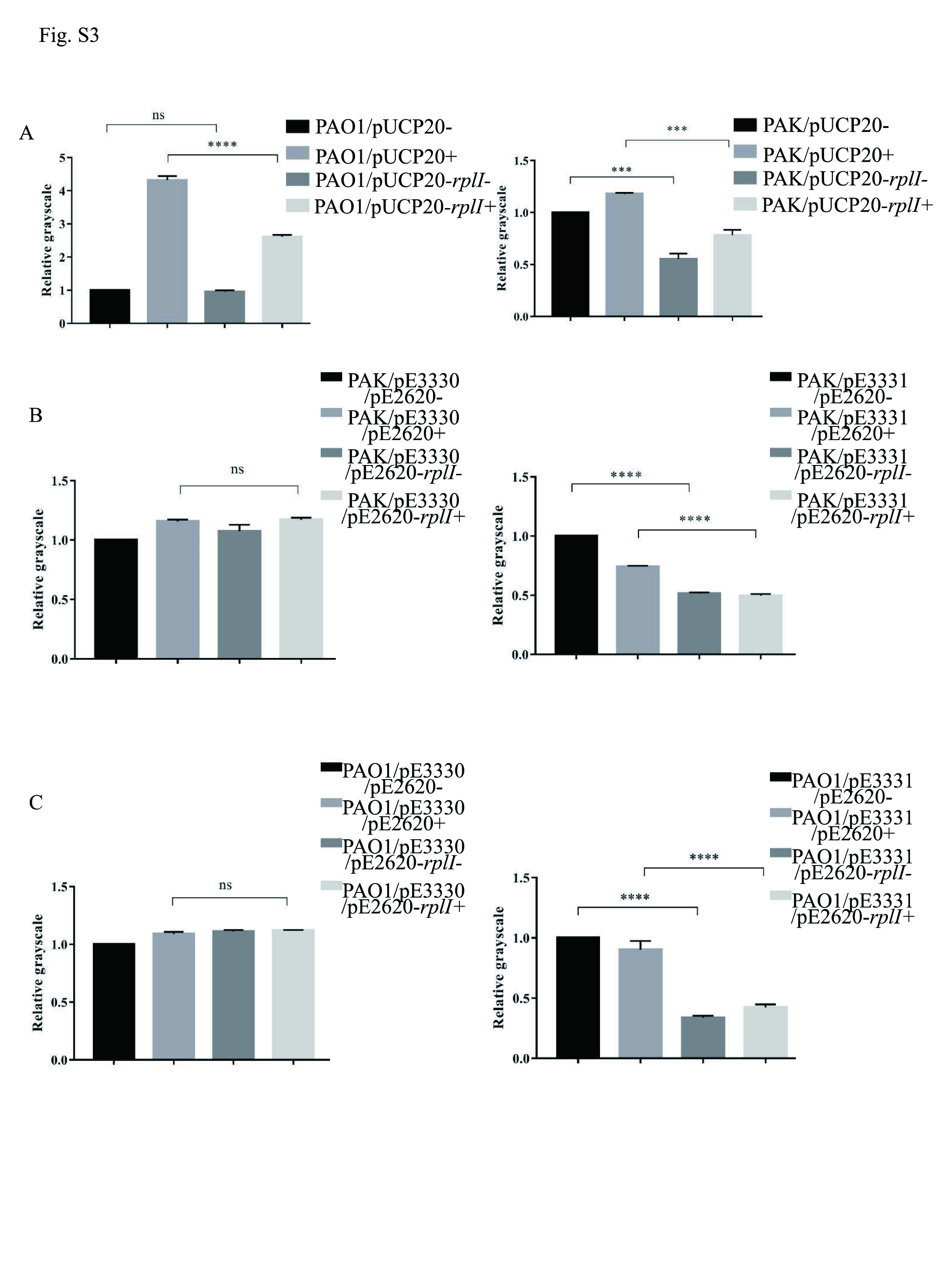

Supplement: S3 Fig — The relative grayscale represents the density of the sample/density of the loading control with the respective first lane as 1. ns, not significant, ***P< 0.001, ****P < 0.0001, by Student’s t test. The data shown represent the results from three independent experiments. (TIF) [file ppat.1010170.s006.tif]

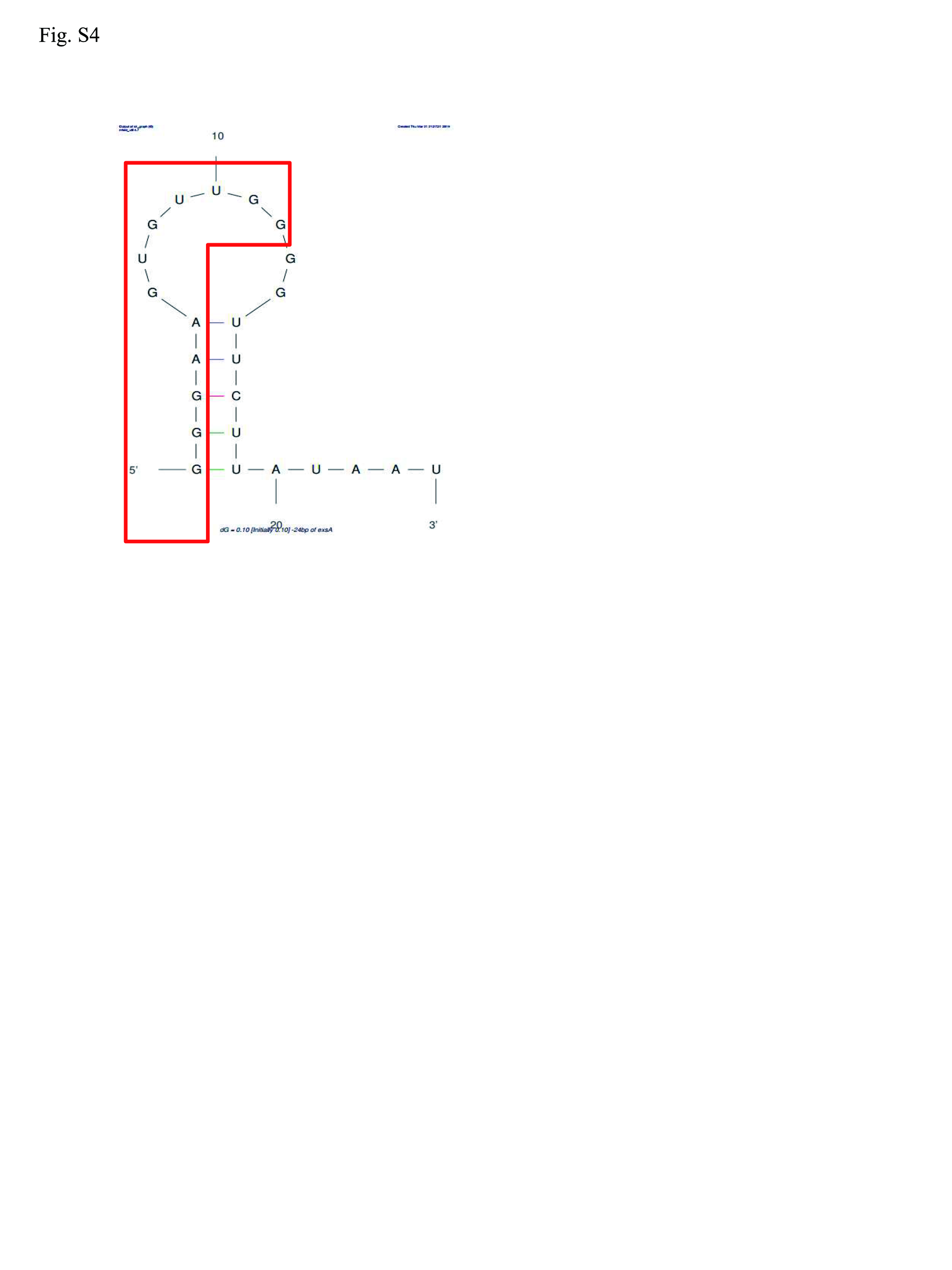

Supplement: S4 Fig — The region from -24 nt to -12 nt was boxed. (TIF) [file ppat.1010170.s007.tif]
